# Immune response in COVID-19: what is next?

**DOI:** 10.1038/s41418-022-01015-x

**Published:** 2022-05-17

**Authors:** Qing Li, Ying Wang, Qiang Sun, Jasmin Knopf, Martin Herrmann, Liangyu Lin, Jingting Jiang, Changshun Shao, Peishan Li, Xiaozhou He, Fei Hua, Zubiao Niu, Chaobing Ma, Yichao Zhu, Giuseppe Ippolito, Mauro Piacentini, Jerome Estaquier, Sonia Melino, Felix Daniel Weiss, Emanuele Andreano, Eicke Latz, Joachim L. Schultze, Rino Rappuoli, Alberto Mantovani, Tak Wah Mak, Gerry Melino, Yufang Shi

**Affiliations:** 1 The Third Affiliated Hospital of Soochow University/The First People’s Hospital of Changzhou, State Key Laboratory of Radiation Medicine and Protection, Institutes for Translational Medicine of Soochow University, Medical College, Suzhou, China; 2 CAS Key Laboratory of Tissue Microenvironment and Tumor, Shanghai Institute of Nutrition and Health, Shanghai Institutes for Biological Sciences/Shanghai Jiao Tong University School of Medicine, University of Chinese Academy of Sciences, Chinese Academy of Sciences, Shanghai, China; 3 Beijing Institute of Biotechnology, Research Unit of Cell Death Mechanism, Chinese Academy of Medical Sciences, 2021RU008, 20 Dongda Street, 100071 Beijing, China; 4 Deutsches Zentrum für Immuntherapie (DZI), Friedrich-Alexander-Universität Erlangen-Nürnberg (FAU) and Universitätsklinikum Erlangen, Erlangen, Germany; 5 Department of Internal Medicine 3 - Rheumatology and Immunology, Friedrich-Alexander-Universität Erlangen‐Nürnberg (FAU) and Universitätsklinikum Erlangen, Erlangen, Germany; 6 Ministry of Health, Rome, Italy; 7 Department of Biology, TOR, University of Rome Tor Vergata, 00133 Rome, Italy; 8 INSERM-U1124, Université Paris, Paris, France; 9 CHU de Québec - Université Laval Research Center, Québec City, QC Canada; 10 Institute of Innate Immunity, University Hospital Bonn, University of Bonn, 53127 Bonn, Germany; 11 Research and Development Center, GlaxoSmithKline (GSK), Siena, Italy; 12 Deutsches Zentrum für Neurodegenerative Erkrankungen (DZNE), Bonn, Germany; 13 Genomics & Immunoregulation, LIMES-Institute, University of Bonn, Bonn, Germany; 14 Department of Biomedical Sciences, Humanitas University, via Rita Levi Montalcini 4, Pieve Emanuele, 20072 Milan, Italy; 15 IRCCS Humanitas Clinical Research Hospital, via Manzoni 56, Rozzano, 20089 Milan, Italy; 16 William Harvey Research Institute, Queen Mary University, London, UK; 17 Princess Margaret Cancer Centre, University Health Network, 610 University Avenue, Toronto, ON M5G 2M9 Canada; 18 Department of Pathology, University of Hong Kong, Hong Kong, Pok Fu Lam, 999077 Hong Kong; 19 Department of Experimental Medicine, TOR, University of Rome Tor Vergata, 00133 Rome, Italy

**Keywords:** Infectious diseases, Antimicrobial responses

## Abstract

The coronavirus disease 2019 (COVID-19) has been a global pandemic for more than 2 years and it still impacts our daily lifestyle and quality in unprecedented ways. A better understanding of immunity and its regulation in response to SARS-CoV-2 infection is urgently needed. Based on the current literature, we review here the various virus mutations and the evolving disease manifestations along with the alterations of immune responses with specific focuses on the innate immune response, neutrophil extracellular traps, humoral immunity, and cellular immunity. Different types of vaccines were compared and analyzed based on their unique properties to elicit specific immunity. Various therapeutic strategies such as antibody, anti-viral medications and inflammation control were discussed. We predict that with the available and continuously emerging new technologies, more powerful vaccines and administration schedules, more effective medications and better public health measures, the COVID-19 pandemic will be under control in the near future.

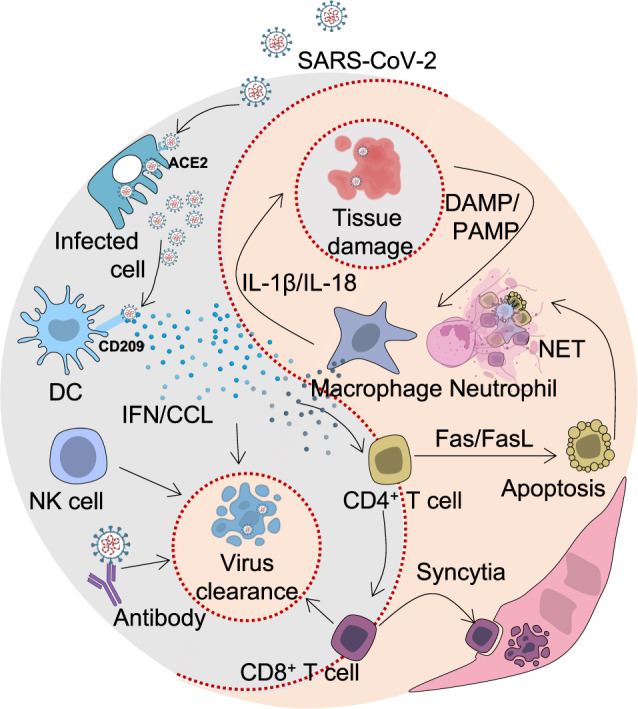

## Facts

1. SARS-CoV-2 infection-associated immune responses are central to the pathogenesis of COVID-19.
2. Innate immune systems sense viral RNA through TLR3, TLR7, and RIG-1 and hyperactivate innate immune responses.
3. Dysregulated neutrophil extracellular traps (NET) formations induce immune-thrombosis and exacerbate inflammation in the lungs of patients with COVID-19.
4. Lymphocytopenia induced by apoptosis and syncytia formation promotes the COVID-19 progression.
5. SARS-CoV-2 vaccines often could not block infection but provide immunity to reduce disease severity.

## Open Questions

1. How to determine the importance of specific CD8^+^ T cells in the immunity to SARS-CoV-2?
2. How will the COVID-19 pandemic end? Will COVID-19 become endemic?
3. How will the Omicron variant evolve? What immune properties will the next variant have?
4. Will herd immunity built up by vaccination and natural infections end the transmission of SARS-CoV-2 virus?

**Fig. 1:**
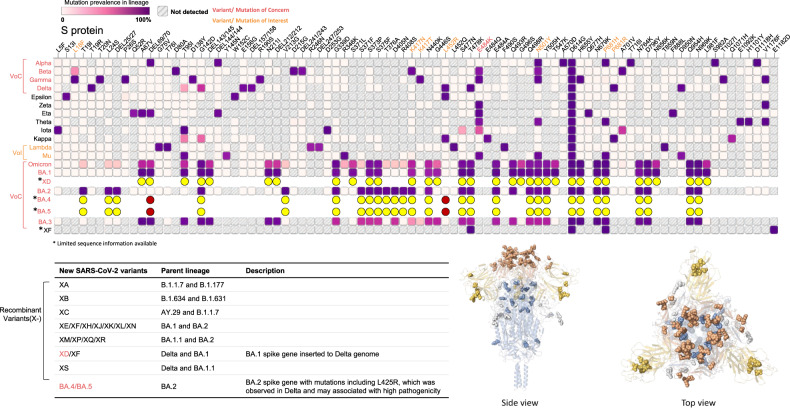
The mutation landscape of the spike proteins of selected SARS-CoV-2 variants. The top panel shows the mutation profiling and prevalence of spike proteins across 13 SARS-CoV-2 lineages that received a Greek designation and 7 recently emerged SARS-CoV-2 variants with public attention. The parent lineages of the new SARS-CoV-2 variants were depicted in the table. The bottom images show the side and top view of the 3-dimension structure for the Omicron spike protein with mutation amino acids mapped [170]. Note: the insertion mutations are not profiled.

Pandemic infectious diseases have wreaked havoc on human society multiple times, including the times of the “Plague of Athens” (over 100,000 deaths in 430 BC), Yersinia pestis (50 million deaths in 1340) or “Spanish influenza” (50 million deaths in 1918). These also include several viral diseases like HIV (40 million deaths in 1980–2000), H1N1 “Swine flu” (300,000 deaths in 2009), yellow fever, Zika, Ebola, SARS, MERS, and the current coronavirus disease 2019 (COVID-19) caused by Severe Acute Respiratory Syndrome CoronaVirus 2 (SARS-CoV-2). Despite that more than two years have passed since the first appearance of COVID-19, the lifestyle, economic activities, and social behaviors of our world are still being impacted by this pandemic [1]. With over 500 million confirmed COVID-19 cases (over 6% of the world population) and circa 6.5 million deaths worldwide, the causing virus, SARS-CoV-2 [2–6], shows a rapidly expanding genealogy to now warranting a classification for at least 13 variants and appears to become endemic, with mutations at the N-terminus and the receptor-binding region, including p.Glu484Lys found in the most dangerous variants [7], Fig. 1. The variants of concern (VoC) have been Alpha, Beta (B.1.351), Gamma (P.1), Delta (B.1.617.2), and Omicron (B.1.1.529), with Delta and Omicron being the most alarming ones [8]. Dreadfully, a new variant with the Delta backbone and Omicron spike has emerged [9]. Great progress has been made in controlling the COVID-19 pandemic, however, much of the efforts still focus on reducing infection and disease severity by vaccination (more than 11 billion vaccine doses administered) [10–12], which occasionally caused some adverse effects [13]. In the meantime, the virus tends to evolve into variants with high transmission and low pathogenicity [14]. Unfortunately, it is almost certain that the virus will gain new mutations, possibly with higher pathogenicity.

Here, we review the yin and yang of innate and adaptive immunity of acute SARS-CoV-2 infection and emphasize open outstanding questions.

## Underlying inflammatory conditions and infection severity

The majority of people infected with SARS-CoV-2 experience mild to moderate respiratory illness, including fever, cough, shortness of breath, muscle aches, headache, loss of taste and smell, sore throat, congestion, or runny nose; while some become seriously ill and require medical attention, especially the elderly and those with underlying medical conditions such as cardiovascular disease, diabetes, chronic respiratory disease, or cancer [15]. Clearly, the inflammatory conditions, as well as the immune status of patients, are critical in determining the course of the disease progression [12].

The deceased among COVID-19 patients exhibited a strong association with age [1]. The group at 30 or younger had fewer mortalities, while the group at 65 or older showed dramatically high mortality (Data.CDC.gov). In most countries, more death was observed in infected men than in infected women [16]. A higher COVID-19 death rate was also observed in smokers, obese individuals, and patients suffering from chronic kidney disease, cardiovascular disease, or cancer [17]. The biggest change in the death rate is associated with the recent appearance of the Omicron variant, which is highly transmissible with a death rate lower than other VoC [18, 19]. Of course, this alteration in the death rate could be due to the success of vaccination. Indeed, it has been reported that among the unvaccinated, especially those over 75-year-old, the mortality is still very significant [20].

As flying mammals, bats are a super zodiac reservoir of viruses, especially coronaviruses. However, bats have a unique immune system that is well balanced between defense and immune tolerance, which prevents them from developing pathological changes after viral infection. They have enhanced constitutive expression of Interferons (IFNs), interferon-stimulated genes, and several heat-shock proteins. On the other hand, bats have reduced stimulator of interferon genes (STING) and suppressed NLR family pyrin domain containing 3 (NLRP3) inflammasome [21]. On contrary to bats, humans are not completely resistant to some coronavirus infection [21]. It is interesting to note that unlike infections with other viruses such as smallpox, measle, or rabies, exposure to SARS-CoV-2, especially with the Omicron variant, of individuals who received a vaccine or recovered from a prior infection with other variants, could result in disease, yet with milder or no symptoms [22]. Such evasion of the immune system makes the elimination of the virus more difficult. Genetic variation in the SARS-CoV-2 virus is certainly a major contributing factor to incomplete immune protection. Most work until now strongly supported the notion that SARS-CoV-2 does not infect circulating blood leukocytes, since they do not express the SARS-CoV-2 receptor, the angiotensin-converting enzyme 2 (ACE2). A very recent study [23] suggested that up to 6% of blood monocytes can be infected with the virus, however, this requires further confirmation. Another important factor is that the mucosal SARS-CoV-2 specific IgM and IgA decay very fast [24]. It is also possible that virus neutralization could only be achieved by the receptor-binding domain (RBD)-specific antibodies and that the RBD is hidden by protein folding until right before binding to ACE2 [25].

## Innate immunity

Numerous studies throughout the last two years have established the innate immune system as a critical defender against SARS-CoV-2. In the best cases, innate immunity eliminates SARS-CoV-2 without activation of the adaptive immune system, thus creating a so-called “never-COVID” cohort. This notion is strongly supported by a recently launched human SARS-CoV-2 challenge study (NCT04865237), in which 36 young health volunteers were intranasally administrated with 10 TCID50 of SARS-CoV-2/human/GBR/484861/2020 (a D614G containing pre-alpha wild-type virus; Genbank accession number OM294022). Surprisingly, 16 volunteers (~44.4%) remained uninfected upon the deliberate SARS-CoV-2 exposure. Their C-reactive protein (CRP), SARS-CoV-2 neutralizing antibody, and spike-specific IgG remain negative, excluding the contributions of adaptive immune cells in such protections [26]. However, the innate immune defenders can also become deleterious when inappropriately activated during SARS-CoV-2 infections [27].

## Cellular innate immunity

Genetic evidence indicates that cell-mediated innate immunity plays a key role in resistance to COVID-19 and in the pathogenesis of severe disease [28–30]. Genes emerging as playing a key role include chemokines and their cognate receptors and members of the IFN pathway. Cellular and innate immune receptors recognizing SARS-CoV-2 belong to different classes [31]. Mouse and human genetic data unequivocally prove that GU-rich RNA sequences are recognized by Toll-like receptor 7 (TLR7) in plasmacytoid dendritic cells (pDC) and TLR8 in conventional DC and myeloid cells [32]. These TLR receptors are located in the endosomal compartment and trigger IFN production (pDC), antigen presentation and uncontrolled inflammation at later stages. Consistent with these in vitro and in vivo mouse data, TLR7 genetic deficiency was associated with severe disease [33]. Cytosolic receptors including the retinoic acid-inducible gene-1 (RIG-1) complex have also been suggested to sense SARS-CoV-2 nucleic acids [31]. Finally, recent evidence suggests that surface C-type lectins interact with the glycosidic components of spike and play an important role in viral entry [34–37].

Pro-inflammatory macrophages are the major immune cell type that expresses high levels of ACE2 [38]. Upon SARS-CoV-2 infection, these macrophages release inflammatory cytokines and chemokines including C-C motif chemokine ligand 7 (CCL7), CCL8 and CCL13 to recruit and activate T cells. In turn, T cells produce IFN-γ and other cytokines to further activate macrophages [39]. This positive feedback loop drives the elevation and continuation of the pathological inflammation. Epidemiological data show that older adults and people with underlying health conditions exhibited a dramatically high rate of severe disease and mortality [17]. Along with aging, there is a tendency of increasing inflammatory macrophages [40]. This not only explains why chronic inflammatory disease occurrence is more prevalent but also provides a possibility accounting for the high incidence of severe COVID-19 cases in older people. Along with this scenario, it is reasonable to comprehend why SARS-CoV-2 infection in those with underlying medical conditions also exhibited a higher prevalence in severe disease and mortality [12].

Single-cell sequencing in combination with cytometry by time of flight (CyTOF), Cite-Sequencing, or multi-color flow cytometry has been particularly informative to describe the deviations of innate immune cells in COVID-19 patients. Early on it was demonstrated that granulocytes and monocytes were dramatically altered in patients with severe disease courses, while moderate and mild disease courses showed rather regular inflammatory cell activation programs with high level human leukocyte antigen-DR (HLA-DR) and CD11c expression [27]. In severe COVID-19, monocytes are characterized by high level expression of alarmins and CD163, while major histocompatibility complex (MHC) molecules are reduced. Within the neutrophil compartment, cell states reminiscent of myeloid-derived suppressor like cells are observed in severe COVID-19 and at the same time cellular programs necessary for neutrophil extracellular traps (NET)-formation are overexpressed. Further, the appearance of neutrophil precursors in the blood is evident for emergency myelopoiesis in patients with severe COVID-19. Mononuclear phagocytes are extremely plastic and diverse and undergo different forms of activation and tolerance [41, 42]. Macrophages’ function has an adaptive component which has been referred to as “training”. Trained innate immunity underlies pathogen agnostic protection associated with selected vaccines, infections and cytokines such as interleukin-1 (IL-1) [43]. There is evidence that trained innate immunity can contribute to resistance against COVID-19. For instance, if mothers were indirectly exposed to live polio vaccine because of vaccination, their babies were found to have decreased symptomatic infection with COVID-19 [44]. The relevance of trained innate immunity to COVID-19 and to vaccines in current use remains to be defined.

A major clinical problem of severe COVID-19 is the development of an “acute respiratory distress syndrome” (ARDS) associated with prolonged respiratory failure and high mortality. Also, here innate immune cells are related to this pathophysiological reaction in severe COVID-19 [45]. In ARDS patients, CD163-expressing monocyte-derived macrophages that acquired a profibrotic transcriptional phenotype accumulate [45]. The profibrotic programs of lung macrophages in COVID-19 are reminiscent of cellular reprogramming previously identified in idiopathic pulmonary fibrosis. Strikingly, the in vitro exposure of monocytes to SARS-CoV-2 sufficiently induced such a profibrotic phenotype [45].

Other innate immune cells are also altered in COVID-19 [46]. For example, in severe COVID-19 patients, Nature killer (NK) cells showed a prolonged expression of IFN-stimulated genes (ISGs), while tumor necrosis factor (TNF)-induced genes were observed in mild and moderate disease. Further, NK cells in severe COVID-19 showed impaired function against SARS-CoV-2 infected cells and impaired anti-fibrotic activity [46]. Other studies suggested that untimely transforming growth factor β (TFGβ) responses limit the antiviral functions of NK cells in severe disease [47]. Surprisingly, other blood-derived cells including megakaryocytes, and erythroid cells were also characterized by an increased expression of ISGs in severe but not mild COVID-19 further supporting prolonged IFN response being directly related to disease severity [48].

Further, SARS-CoV-2 seems to trigger an innate functionality in a subset of T cells, namely highly activated CD16^+^ T cells, which occur mainly in severe COVID-19 in the CD4, CD8 and γδ T cell compartments [49]. It was demonstrated that increased generation of C3a in severe COVID-19 induced this peculiar T cell phenotype. Functionally, CD16 enabled immune-complex-mediated, T cell receptor (TCR)-independent degranulation and cytotoxicity, which so far, seems to be specific to SARS-CoV-2. These functions were further linked to the release of neutrophil and monocyte chemoattractants and microvascular endothelial cell injury, the latter being made responsible for the heterogeneous and manifold clinical symptoms involving many different organs in severe COVID-19. Worrisome is the persistence of the cytotoxic phenotype of CD16^+^ T cell clones beyond acute disease which might also be involved in pathophysiological mechanisms associated with long COVID. However, this clearly requires further investigation. Innate functionality of CD16^+^ T cells not only seems to play an important pathophysiological role, but the proportion of these cells together with plasma levels of complement proteins upstream of C3a were shown to be associated with fatal outcomes.

## Humoral innate immune response to SARS-CoV-2 infection

Innate immunity consists of a cellular and a humoral arm [50]. Components of the humoral arm of innate immunity are a diverse set of molecules, such as Complement components, collectins (e.g., Mannose-binding lectin, MBL), ficolins, and pentraxins (e.g., C reactive protein, CRP, and PTX3) [50, 51]. These fluid phase pattern recognition molecules have functions similar to antibodies (ante-antibodies). Among these ante-antibodies, MBL was found to bind spike by recognizing its glycosidic moieties and to inhibit SARS-CoV-2 [36]. All VoCs including Omicron were recognized by MBL. MBL haplotypes were found to be associated with disease severity [36]. Pentraxin 3 (PTX3), but not its distant relative CRP bound the SARS-CoV-2 nucleoprotein, but it remains to be elucidated whether its recognition amplifies inflammation [36]. Indeed, PTX3 has emerged as an important biomarker of disease severity with for instance death as the endpoint [52–56]. The results have been extended to long COVID [57] with PTX3 being part of a disease severity signature.

Complement has emerged as a pathway of amplification of inflammation and tissue damage [58]. The lectin pathway may play a role in complement activation. Small pilot studies suggest that targeting complement by inhibiting the C3 convertase or by blocking mannose-associated serine protease (MASP) and the lectin pathway may be beneficial in COVID-19 [49, 59–63]. Whether these therapeutic approaches might also impact the functionality of the highly activated CD16^+^ T cells with innate immune function requires further investigation [49].

Thus, humoral innate immunity (ante-antibodies) plays an important role in COVID-19. MBL represents a non-redundant pathway of resistance against SARS-CoV-2 VoC. The pentraxins CRP and PTX3 provide important prognostic indicators, with PTX3 integrating myeloid cell and endothelial cell activation. It will be important to further explore the value and significance of ante-antibodies as biomarkers (PTX3), candidate therapeutics (MBL) and therapeutic targets (complement).

Macrophages and monocytes express a variety of pattern recognition receptors (PRRs), including TLRs, (NOD)-like receptor family proteins (NLRs), absent in melanoma 2 (AIM2) and the cyclic GMP-AMP synthase (cGAS)-STING pathway. These can trigger innate immune responses to viral infection through direct infection and sensing of SARS-CoV-2 or by detecting damage-associated molecular patterns (DAMPs) or pathogen-associated molecular patterns (PAMPs) released by infected cells that act as a feedforward mechanism propagating the systemic inflammatory response.

Single-cell sequencing and flow cytometric analyses have established the presence of SARS-CoV-2 RNA in human lung macrophages [30, 39] and blood monocytes [64]. Neither human lung macrophages nor monocytes express the primary SARS-CoV-2 internalization receptor ACE2, and as such alternative mechanisms for viral internalization have been proposed, including Fc-receptor mediated uptake [23, 65]. Lung myeloid cells infected with SARS-CoV-2 induce the transcriptional programs and signaling cascades of innate immune response. SARS-CoV-2-infected cells upregulate chemokines, cytokines, IFN pathway and TNF associated genes [30, 39]. These act to inhibit viral expansion and recruit monocytes and T cells to the site of infection. However, excessive release of pro-inflammatory cytokines was early identified in severe COVID-19 patients [66]. The detection of viral RNA potentially drives activation of this transcriptional response by endosomal TLR3 and TLR7, as well as SARS-CoV-2 E protein detection on the cell membrane by TLR2 [67].

Recent reports have shown the presence of an oligomerized apoptosis-associated speck-like protein containing a caspase-activating and recruitment domain (CARD) alongside NLRP3 in monocytes and lung macrophages from COVID-19 patients [68]. The monocytes displayed a concomitant activation of caspase-1, and cleavage and translocation of the gasdermin D pore complex to the plasma membrane, a downstream event of inflammasome activation that facilitates cytokine release and precedes the inflammatory lytic cell death process known as pyroptosis. Indeed, sera from COVID-19 patients are enriched for IL-1β, IL-18 and lactate dehydrogenase (LDH), indicative of ongoing pyroptosis [68]. In the lungs of COVID-19 individuals, inflammasome activation is not exclusive to SARS-CoV-2-infected cells, suggesting that paracrine signals caused by SARS-CoV-2 infection can induce pyroptosis in neighboring cells, potentiating the inflammatory response and disease severity [64].

Inhibitors targeting inflammasome pathway components, including caspase-1 and NLRP3 reduced pathology in a humanized mouse model of SARS-CoV-2 infection [65], suggesting therapeutic targeting of the NLRP3 inflammasome may provide translational benefit as society proceeds to live alongside SARS-CoV-2. However, it is important to bear in mind that most studies on human patients rely on post-mortem tissues and therefore represent the most severe form of the disease. Consequently, it remains to be seen whether inflammasome inhibition can yield effective results in mild forms of COVID-19.

The innate immune response to SARS-CoV-2 infection is not limited to macrophages and monocytes, and is frequently associated with abnormal activation and recruitment of neutrophils. It has been reported that there is a dramatic increase in myeloid-derived suppressor-like cells (MDSC-like) [69], particularly in those at severe stages of COVID-19, contributing to the pathogenesis of SARS-CoV-2 infection. MDSCs may delay the clearance of the SARS-CoV-2 virus and inhibit T cell proliferation and functions. Neutrophils are known to release NET and the imbalance between NET formation and degradation plays a central role in the pathophysiology through trapping inflammatory cells and preventing the recruitment of tissue repairing cells. Strategies that dysregulate the formation of NET or destruct NET with agents such as DNase could represent new therapies for COVID-19 patients, especially those suffering from severe illness [2], see below.

## NET-driven vascular occlusions drive pathology in severe COVID-19

During the membrane rupture of granulocytes in the process of NET formation the preformed pro-inflammatory cytokines (e.g., IL-6) and chemokines (e.g., IL-8, CCL3) as well as antimicrobial peptides (e.g., bactericidal/permeability-increasing protein and histones), serine proteases (e.g., neutrophil elastase and proteinase 3), other enzymes (e.g., myeloperoxidase, lactoferrin, lysozyme and phospholipase A2), and reactive oxygen species (ROS) are released into the vicinity of NET. The activity of the soluble mediators fades as NET formed in high neutrophil densities tends to aggregate. These aggregates act anti-inflammatorily as NET-borne proteases proteolytically degrade inflammatory mediators and toxic histones [70, 71]. Importantly, DNA-bound proteases are not antagonized by anti-proteases [72]. Thus, the formation of NET is considered a double-edged sword that initially initiates inflammation and later helps to orchestrate its resolution.

**Fig. 2:**
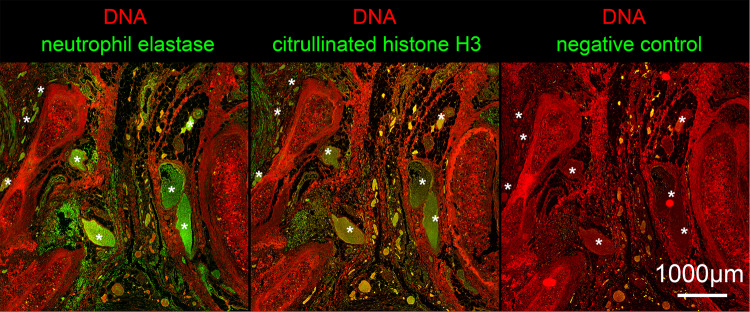
NETosis. Immune fluorescence detects the NET components citrullinated Histone H3 and neutrophil elastase (both green) as well as extranuclear DNA (DAPI; red) in the vessels of a central human lung. Note, the intravascular DNA-enzyme-histone complexes fill the whole lumen of many vessels (some of the clogged vessels are marked with asterisks).

The imbalance between NET formation and degradation can also drive inflammation, e.g., by occluding vessels and ducts [73]. First reports about the role of NET in patients with COVID-19 were already published soon after the onset of the pandemic describing elevated levels of NET markers such as cell-free DNA, citrullinated Histone H3 (citH3), and myeloperoxidase-DNA (MPO-DNA) complexes in the sera of these patients [74]. Single cell sequencing of blood-derived neutrophils from peripheral blood supported a reprogramming of a subset of neutrophils towards NET formation-related transcriptional programs especially in severe COVID-19 [27]. Serum from patients with COVID-19 as well as the virus itself were reportedly able to trigger NET formation accompanied by increasing levels of intracellular ROS [74–76]. This ROS-NET pathway together with the activation of neutrophils, the formation of neutrophil-platelet aggregates, and intravascular aggregation of NET enriched with complement and tissue factors form occlusive NET-derived immunothromboses, Fig. 2. This is particularly dangerous in the microvasculature, where severe organ damage occurs due to disrupted microcirculation [72, 77–79]. Because of their central role in the pathophysiology of COVID-19, NET is a prime target for therapeutical intervention. Therapeutic doses of heparin were shown to prevent the aggregation of NET by nano- and microparticles and the efficiency of this therapy in COVID-19 patients was shown recently [80, 81]. Furthermore, Heparin is known to accelerate the DNase I-mediated degradation of NET and first trials with Dornase Alfa, a recombinant DNase, have been undertaken [82, 83]. Disulfiram was also reportedly successful in the reduction of NET, increase of survival, and improvement of blood oxygenation in animal models, which makes it a new promising candidate for the treatment of NET-related pathologies in patients with COVID-19 [84]. Lastly, inhibitors of peptidyl-arginine deiminases (PADs) are discussed as therapies to treat NET-related thrombotic complications in patients with COVID-19, however, no clinical trial has been conducted yet [85].

## Targeting the type I IFN production by SARS-CoV-2

While innate immunity constitutes the first line of host defense against virus infection, the type-I IFN response is the core that endows antiviral activities to host cells, which consists of two major consecutive steps including IFN production and the expression of ISGs [86]. Here, we specifically focus on the regulation of type-I IFN production, which is the first and critical step for an effective innate immune response and therefore is primarily targeted by SARS-CoV-2 proteins for suppression.

**Fig. 3:**
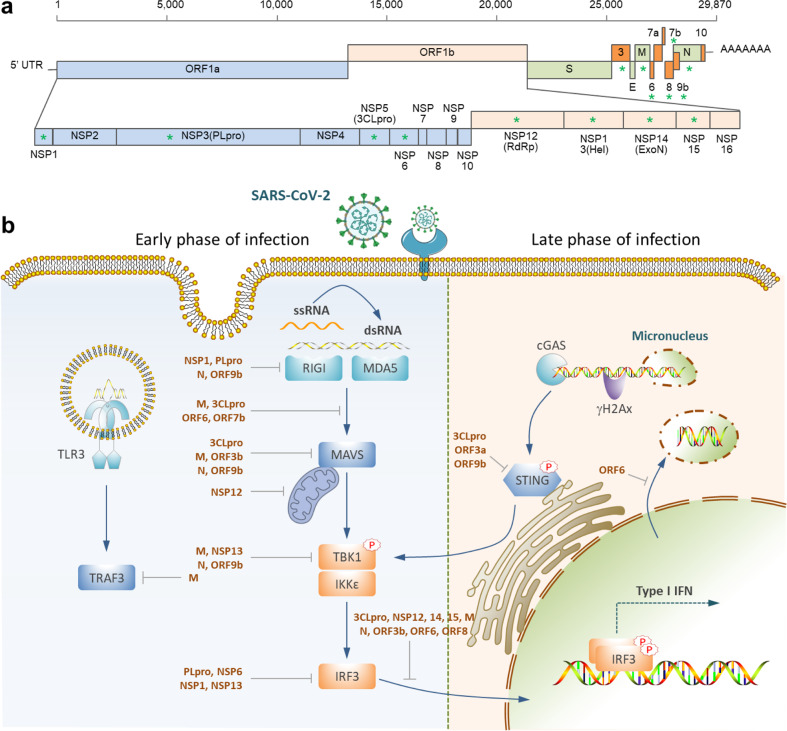
Targeting type I IFN production by SARS-CoV-2. **a** Schematic demonstration of the viral proteins. Those marked with asterisks were reported to regulate IFN production. **b** IFN production signaling pathways targeted by SARS-CoV-2 proteins. SARS-CoV-2 infection induces a delayed type-I IFN response, which is underlaid by the inhibited RIG-I/MDAS-MAVS signaling at the early stage and the cytoplasmic-micronuclei-activated cGAS-STING signaling at the late stage.

As depicted in Fig. 3, the type-I interferon production is initiated by the recognition of the double-strand RNA (dsRNA) generated during the virus life cycle by the RIG-1-like receptors (RLRs), including the RIG-1 and/or melanoma differentiation gene 5 (MDA5) in the cytoplasm, or the TLRs in the endosome [87]. Upon loading with dsRNA, RIG-1 and MDA5 can interact with the adapter mitochondrial antiviral signaling protein (MAVS), leading to the formation of a signaling complex consisting of TANK-binding kinase 1 (TBK1) and inducible IκB kinase (IKKi). The TBK1/IKKi complex then phosphorylates interferon regulatory factor 3/7 (IRF3/7), promoting their translocation into the nucleus to drive IFN-α/β expression. Meanwhile, the TLRs, such as TLR3, could also recognize PAMPs in the endosome to induce cytokines and chemokines production, enhancing the innate immune response [87].

During this process, it was reported that SARS-CoV-2 encoded at least 14 proteins, accounting for about half of the total proteins encoded by the virus, to interfere with IFN production [88–90]. These proteins include the structural membrane (M), nucleocapsid (N) proteins, the accessory proteins (3, 6, 8, and 9b), and the nonstructural proteins (NSP1, 3, 5, 6, 12,13, 14, and 15) generated from a large open reading frame (ORF) encoding 1ab by papain-like proteinase (NSP3, NLpro) and 3C-like proteinase (NSP5, 3CLpro) -mediated cleavage. Suppression of IFN production by the SARS-CoV-2 proteins was primarily executed through four types of mechanisms, including escaping viral RNA recognition (by N, ORF9b, NSP1, and NLpro), compromising RIG-1 or TLRs signaling (by M, N, 3CLpro, NSP12, ORF3b, ORF6, ORF7b, and ORF9b), targeting the TBK1 complex (by M, N, NSP13, ORF9b,), and interfering with IRF3 activation (by M, N, NLpro, 3CLpro, NSP1, NSP12–15, ORF3b, ORF6, and ORF8), Fig. 3. Corresponding to the extensive interference of IFN production by SARS-CoV-2-encoded proteins, patients with COVID-19 usually exhibited a delayed type-I IFN response [90], i.e., IFN production was inhibited at the early stage of SARS-CoV-2 infection, which allows the virus to achieve successful replication in the host cells, undermining the asymptomatic infection. Enhancing IFN response at this stage turned out to help restrict SARS-CoV-2 infection [91–93].

Subsequent to a latent IFN response, the patients with COVID-19, particularly those in severe forms, exhibited a substantially exaggerated IFN response manifested with uncontrolled cytokine storm and inflammation, corresponding to another arm of the delayed type-I IFN response at the late stage [90], on which recent studies shed lights. Zhao et al. reported that an expression level-based dual-role of the structural N protein may be partially accountable, where the low-dose N protein was suppressive while the high-dose was promotive, for the activation of IFN signaling. This worked out by dually regulating the phosphorylation and nuclear translocation of IRF3 [94]. Alternatively, Ren et al. found that SARS-CoV-2 may activate IFN response unexpectedly via the cGAS-STING signaling pathway, which was induced by the cytoplasmic micronuclei produced in the multinucleate syncytia between cells expressing spike and ACE2 [95]. And the results were further confirmed independently by Zhou et al., who demonstrated that cell-cell fusion was sufficient to induce cytoplasmic chromatin, and the cytoplasmic chromatin-cGAS-STING pathway, but not the MAVS-mediated viral RNA sensing pathway, contributes to interferon and pro-inflammatory gene expression upon cell fusion [96]. Interestingly, several SARS-CoV-2 proteins (3CLpro, ORF3a, and ORF9) were also able to target STING to regulate the IFN response [97], likely indicating a complex feedback interaction between SARS-CoV-2 and the innate immunity, and therefore a well-balanced immune interference targeting IFN response is required for COVID-19 therapy.

## Adaptive immunity: humoral immunity to SARS-CoV-2

**Fig. 4:**
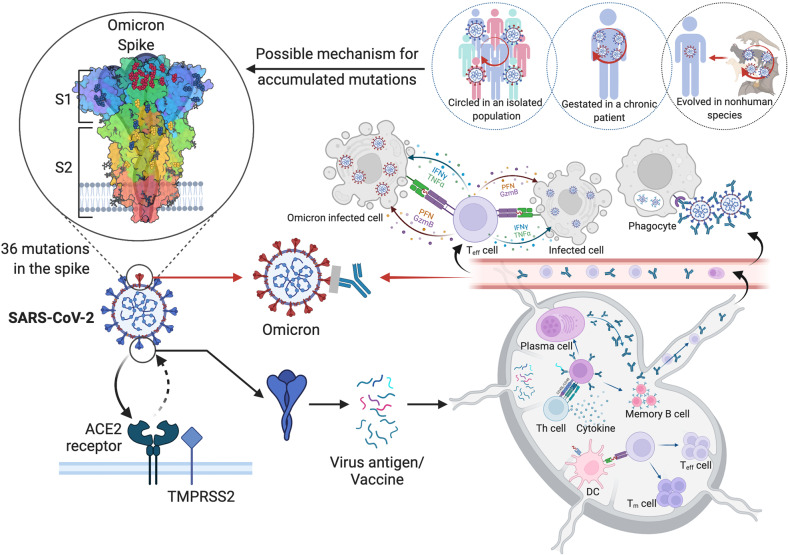
The SARS-CoV-2 Omicron variant with high mutational burden exhibits increased antibody evasion. In a typical SARS-CoV-2 infection, the virus presented in the lymphoid organs evokes T helper cells which facilitate the activation of both humoral and cellular immune responses. Antibodies and effector CD8^+^ T cells were then released into circulation. Antibodies neutralize virus and eliminate infected cells through ADCC. CD8^+^ T cells kill infected cells through cytotoxicity. However, as for SARS-CoV-2 Omicron, the effects of antibody-mediated protection were dramatically reduced, which is possibly brought about by over 30 mutations in the genes encoding spike proteins. There are 3 possibilities explaining the sudden appearance of Omicron: 1. Omicron may have been transmitted within a neglected population without sufficient medical surveillance; 2. It could be outcompeted in a patient with chronic COVID-19 infections; 3. It may be zoonotic and just spilled back into human.

Adaptive immunity provides pathogen-specific immunity, which eradicates infection and provides long memory and recall of the immune responses, Fig. 4. By producing antibodies, B cells play a critical role in anti-viral immunity. Different classes of antibodies such as IgM, IgA, IgG, and IgE, are involved in humoral immune responses to viral infections. These antibody classes are characterized by their intrinsic properties, functions, tissue distributions, and half-lives. Upon SARS-CoV-2 infection or vaccination, IgD and IgM are the first antibody types produced. The positive test of IgM antibody indicates that the virus may be present or a patient recently recovered from the infection and that the virus-specific immune response has begun [98]. During SARS-CoV-2 infection, symptoms start around day 5 and the body begins to produce IgM antibodies around 7–8 days post-infection [99]. Due to inadequate affinity maturation, IgM antibodies have a relatively low affinity compared to IgG. On the other hand, due to their pentameric nature, IgM antibodies have high avidity for antigens and play critical roles in opsonization.

IgG antibodies usually appear later during an immune response because of the time needed for their affinity maturation to acquire high avidity and more potent capacity to neutralize pathogens, activate the complement pathway, and kill infected cells through antibody-dependent cellular cytotoxicity (ADCC). IgG antibodies have a relatively long half-life in serum and are associated with B cell memory. IgG antibodies to SARS-CoV-2 do not develop until around 14 days post-infection [100]. A positive test for IgG is a good indication of having been infected or vaccinated. Interestingly, among those infected by SARS-CoV-2, detectable IgG antibodies are mainly IgG1 & IgG3 [101].

IgA antibodies are produced right following IgM with serum levels higher than IgM and are the main antibody class in mucosal surfaces and secretions. It has been reported that the SARS-CoV-2-specific IgA can be detected prior to the appearance of IgM and dominates the early neutralizing responses [102]. IgA forms dimers upon secretion to increase avidity. IgA antibodies secreted into the respiratory tract play a key role in mucosal immunity to SARS-CoV-2 infection by facilitating aggregation and preventing the initial infection of host cells. It is important to note that detectable levels of neutralizing antibodies against SARS-CoV-2 start declining within three months following mild and asymptomatic infections. This might predict transient immunity and heightened risk of reinfection.

Intriguingly, several groups have reported a clear association between the extent of T cell immunity and humoral response in convalescent individuals [103–105]. Patients with severe COVID-19 were found harboring low mutation frequencies in their heavy-chain variable region genes in the early weeks after infection, notably in those antibodies against the spike protein [106], indicating suboptimal immunoglobulin maturation. Furthermore, a delay in the emergence of antibodies, including the anti-SARS-CoV-2 neutralizing antibodies, has also been noted in severe forms compared with milder forms of COVID-19 [107, 108]. These are in line with the fact that CD4^+^ T cells are essential for sustaining germinal center (GC) formation and B cell differentiation leading to isotype switch and immunoglobulin maturation, two features of T cell-dependent humoral response. Consistently, defective GC formation is associated with CD4^+^ T cell depletion in the lymph nodes of severe COVID-19 patients [109]. This defect and delay to develop antibodies against spike protein may contribute to viral dissemination and longer persistence of SARS-CoV-2 in patients [110]. Moreover, premature T cell depletion due to apoptosis was associated with a lower B-cell response in individuals infected with filovirus [111] or retrovirus [112, 113]. Therefore, to what extent whereby the death of CD4^+^ T cells by apoptosis [114] may correlate with a delay in mounting an efficient humoral response and in developing sequelae merits further investigation.

## Adaptive immunity: cellular immunity and resistance to SARS-CoV-2

Specific cellular immunity to SARS-CoV-2 is mediated by T cells. These cells are naïve and circulate in the bloodstream and peripheral lymphoid organs until encountering their specific antigen peptide presented by MHC. The SARS-CoV-2 virus itself or naked viral peptides could not activate T cells. High-affinity interaction between self MHC presented SARS-CoV-2 peptides and TCR induces proliferation and differentiation of T cells into cells capable of contributing to the removal of virus-infected cells or helping the production of antibodies. Class I MHC presented endogenous antigen peptides that activate CD8^+^ T cells, while class II MHC presented exogenous antigen peptides that activate CD4^+^ T cells. SARS-CoV-2 specific T cells are critical in the immunity to infection and susceptibility to severe disease has been reported to correlate with HLA alleles [115]. It is known that inflammatory monocytes and macrophages as well as DC express ACE2 which permits the entry of SARS-CoV-2 into these professional antigen-presenting cells to activate T cells, especially CD8^+^ T cells. Although the ACE2 expression on macrophages and DC is only at intermediate levels, the co-expression of CD209 (DC-SIGN) could dramatically facilitate SARS-CoV-2 entry into DC [116]. It should be pointed out that in most cases, SARS-CoV-2 infections do not elicit a dramatic inflammatory response in macrophages and DC. IL-6 is almost undetectable and other cytokines such as IL-1 are very low [117]. This may limit their migration to local lymphoid tissue and maturation to cells with the expression of co-stimulation molecules that are highly effective at presenting antigen to recirculating T cells, indicating a robust T cell response to SARS-CoV-2 may be difficult to induce and thus limit the development of immunity. It is important to note that B cells can also serve as SARS-CoV-2 antigen-presenting cells, especially those with surface immunoglobulin specific to SARS-CoV-2 antigens.

Patients with COVID-19 at the severe stage were manifested with decreased peripheral lymphocytes, termed lymphopenia or lymphocytopenia, which was believed to promote the disease progression [118]. Since lymphocytes barely express ACE2, it is unlikely to be a direct target of the SARS-CoV-2 virus [119]. Two major indirect mechanisms were proposed to account for lymphocyte loss. One is the enhanced cell-autonomous death, primarily by apoptosis. T cells isolated from patients with severe COVID-19 exhibited an increased propensity to die via apoptosis, as evidenced by a higher level of caspase activation and phosphatidylserine exposure, and a high rate of spontaneous apoptosis [114]. This was strongly associated with increased soluble Fas ligand in sera, and with increased expression of Fas/CD95 on T cells, particularly on CD4^+^ T cells [114, 120]. While both extrinsic and intrinsic apoptosis, but not necroptosis, were found to be involved, treatment with Q-VD, a pan-caspase inhibitor, protected the isolated T cells from cell death and enhanced the expression of Th1 transcripts [114]. Consistently, TNF-α and IFN-γ were found prominently upregulated in the sera of patients with severe COVID-19, which was related to a phenomenon called cytokine storm or also viral sepsis [31]. This phenomenon is at least in part induced by an inflammatory cell death PANoptosis, abbreviated for the mixed cell death of pyroptosis, apoptosis, and necroptosis. Importantly, the TNF-α/IFN-γ-induced lethality in animals could be rescued, along with the increased level of T cells, in *Ripk3*^*‒/‒*^*Casp8*^*‒/‒*^ mice, in which PANoptosis was suppressed [121].

Another mechanism responsible for lymphocyte loss is non-autonomously mediated by syncytia, which could be efficiently induced by SARS-CoV-2 via its fusogenic spike protein dictated by an embedded bi-arginine motif [122]. The multinucleated syncytia were found to be able to internalize infiltrated live lymphocytes, preferentially the CD8^+^ T cells, to form cell-in-cell structures, a unique phenomenon that is prevalent in tumor tissues [123] and plays important roles in clonal selection and immune homeostasis and the like [124–126]. By defaults, as taking place in cancer cells, the formation of the cell-in-cell structure primarily resulted in the death of the internalized lymphocytes within syncytia, leading to a rapid elimination, which could be rescued by either blocking syncytia formation or cell-in-cell-mediated death, thus providing a novel target for COVID-19 therapy [127–130]. Interestingly, the two lymphopenia mechanisms seem to exhibit a preference for the targeted lymphocytes, with the autonomous one for CD4^+^ T cells while the non-autonomous one for CD8^+^ T cells [114, 122], whether the preference also applies to other types of lymphocytes and its biological properties and potential implications warrant further investigation. In addition to directly regulating the cellularity of lymphocytes, syncytia were recently shown to be able to activate the cGAS-STING signaling via inducing the naked cytoplasmic micronuclei [95, 96], and to initiate inflammatory cell death [131], both of which, though may help mount an anti-infection immunity, would eventually promote inflammation and tissue damages leading to severer clinical conditions. Coincidently, the less pathogenic Omicron variant of SARS-CoV-2 displayed a compromised ability to induce syncytia formation in cells expressing human ACE2 [132–134]. Together, syncytia and various types of cell death may serve as an important hub for pathogenesis induced by SARS-CoV-2 infection.

Though it is generally recognized that T cell immunity plays a central role in the control of SARS-CoV-2, its importance is still underestimated and mechanistically unclear. A proper T cell response is important to limit infection. Unlike antibodies, which are less sustained and only those specific to RBD can neutralize, T cells react to at least 30 epitopes of viral proteins and exhibit sustained memory. That CD4^+^ T cells are more prone to undergo apoptosis may also contribute to the development of “helpless” CD8^+^ T cells, which are exhausted and shorter-lived cells [135, 136], leading to defective T cell toxicity [137] and death of CD8^+^ T cells [114] in patients with severe COVID-19. In addition, the aforementioned highly activated CD16^+^ T cells also contribute to the pathophysiology of COVID-19 [49]. Thus, preventing lymphopenia, the death of T cells, and the inappropriate functionality of CD16^+^ T cells could be of interest to limit pathogenicity and probably long-term sequela. Consistently, the use of caspase inhibitor in the early phase of infection has provided protection for monkeys developing acquired immunodeficiency syndrome (AIDS) [138]. Therefore, similar strategies might be of interest for SARS-CoV-2 infection. Meanwhile, although COVID-19 in children is rarely severe, a subset of patients developed multisystem inflammatory syndrome in children with robust type II interferon and NF-kB responses, manifested by a transient expansion of TRBV11-2 T cell clonotypes and signs of inflammatory T cell activation [139]. An association with HLA A*02, B*35 and C*04 alleles suggests a genetic predisposition, yet to be validated in larger cohorts [139].

Whilst physical and mental stresses, either acute or chronic, have a dramatic impact on the immune system, both innate and adaptive immune components could be affected. Humans and animals subjected to stress conditions exhibited significant reduction in lymphocytes [140], increase in IL-6 production [141], decrease in IFN-γ [142], augmentation in regulatory T cells [142], and alteration in gut microbiota [143]. The induction of Fas expression on lymphocytes by chronic stress [144] could account, at least in part, for the lymphopenia development in COVID-19 patients mentioned above. It is imperative to emphasize that the prevalence of stress associated with anxiety, depression, fear, and inadequate social support during the COVID-19 pandemic should not be ignored. There is a strong need to understand the stress impact on COVID-19 experiences and stress management should be included in the care of patients, especially those suffering from mental and psychological disorders.

## Therapeutic antibody against SARS-CoV-2

Monoclonals, whether of animal or human origin, have been used for the development of most non polymerase chain reaction (PCR)-based diagnostic tests. They usually target the nucleocapsid, which is the most abundant protein of the virus. During the pandemic, they have been used in hundreds of millions of rapid tests. Herein, we will focus our attention on the use of human monoclonal antibodies (hmAbs). Before the SARS-CoV-2 pandemic, hmAbs had been widely used to treat cancer, inflammatory and autoimmune diseases [145]. With the exception of an antibody against the respiratory syncytial virus approved for clinical use in 1998 [146], hmAbs had not been used for infectious diseases because they require large quantities to be delivered intravenously and were too expensive compared to infectious disease standard of care. The game started to change with a pioneering work done by Antonio Lanzavecchia during the 2002–2003 outbreak of SARS-CoV-1 [147]. For the first time, his lab was able to clone from a convalescent patient a B cell producing antibody neutralizing the virus. Since then, many other technologies became available to isolate hmAbs starting from the B cells of convalescent or vaccinated donors. Many antibodies were developed and tested in the clinic for HIV, and the improved technology allowed to isolate antibodies that were more than 1000-fold more potent of the ones initially isolated against this pathogen [148]. In this environment, the Wellcome Trust published in 2019 a report stating that the time to develop hmAbs for infectious diseases was mature (Wellcome Trust. “Expanding Access to Monoclonal Antibody-Based Products.” (2020)).

**Fig. 5:**
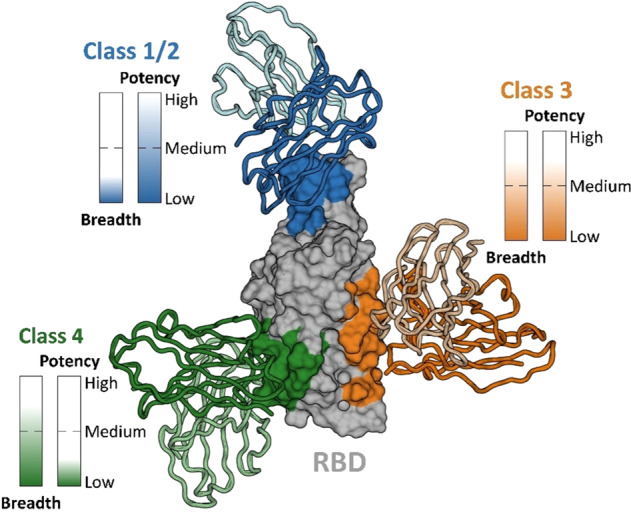
The figure shows the SARS-CoV-2 spike protein receptor-binding domain (RBD) bound by class 1/2 (blue), class 3 (orange) and class 4 (green) neutralizing antibodies. The potency and breadth of neutralization across SARS-CoV-2 variants are denoted for each antibody class [171–173].

As soon as the SARS-CoV-2 pandemic started, multiple academic and industrial laboratories isolated B cells from convalescent people and generated numerous publications in prestigious journals showing the identification of hmAbs able to neutralize the virus in vitro, as well as protecting and treating mice, hamsters, and non-human primates in vivo from viral challenge [149]. The neutralizing antibodies are usually divided into four classes which bind different regions of the spike, Fig. 5. In addition to the pre-clinical evidence, several clinical studies showed that, when used early after infection, hmAbs had very high efficacy in preventing severe disease [150]. Given their efficacy, and since these were the first therapeutic molecules developed during the pandemic, numerous hmAbs received emergency use in the US and Europe: REGN COV2 (Casirivimab and Imdevimab), Bamlanivimab (LY-CoV555), Sotrovimab (VIR 7831 or S309), Evusheld (tixagevimab and cilgavimab) and Bebtelovimab (LY-CoV1404) [150]. For more than one year, hmAbs remained the only real therapeutic tool we had against SARS-CoV-2. Unfortunately, hmAbs did not work in a therapeutic setting during advanced severe disease in hospitalized patients and many of them fell short with the emergence of SARS-CoV-2 variants carrying different mutations on the spike protein, the major target for neutralizing hmAbs. In fact, with the emergence of the SARS-CoV-2 Omicron variant, 85% of hmAbs approved for clinical use lost their potency against this virus [151]. Today we still have three approved hmAbs that work reasonably well against Omicron and new potent monoclonals against this variant are described in the literature [151]. In conclusion, the COVID-19 pandemic has suggested prevention and therapeutic potential of hmAbs to infectious diseases and that can be developed faster than any other medicine. In addition, it is now possible to develop extremely potent hmAbs that can be administered intramuscularly rather than intravenously, facilitating their administration outside the hospital. Therefore, hmAbs can be considered at the forefront of medical interventions in the field of infectious diseases as their characteristics make them essential tools to tackle emerging pathogens and pandemics.

## The SARS-CoV-2 vaccination

**Fig. 6:**
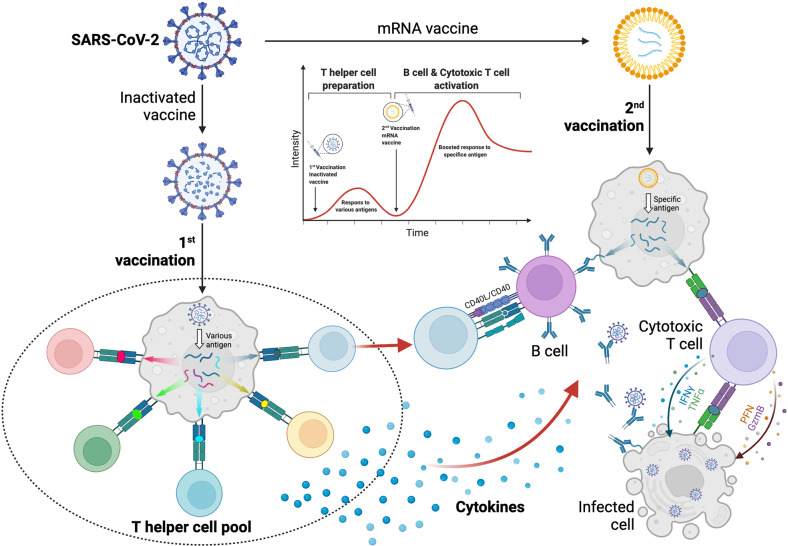
Heterologous prime-boost strategies with inactivated vaccine (1^st^) and mRNA vaccine (2^nd^) provide strong protections against SARS-CoV-2. Inactivated SARS-CoV-2 vaccine reserves all viral proteins for immune recognition. Once immunized, these antigens could elicit a T helper pool broadly targeting SARS-CoV-2 proteins. mRNA vaccine, on the other hand, elicits strong humoral and cellular immune responses against the SARS-CoV-2 variants in individuals who previously received the inactivated vaccine. We hypothesized that the T helper pool primed by inactivated vaccine could be activated upon mRNA vaccination, which facilitates the building of stronger immune response and memory.

Exposure to attenuated pathogens or parts of the pathogens to induce specific immunity against a pathogen started with smallpox variolation in China more than 1000 years ago [152]. Edward Jenner employed cowpox to protect humans from smallpox 800 years later. Since in Latin the word Vacca is for cow, the term vaccination is adapted later by Jenner’s friend Richard Dunning in 1800. In the last 200 years, various approaches have been developed to shelter human beings from different infections through vaccination. One of the most impressive results of modern medicine is the development of highly effective vaccines against SARS-CoV-2 in less than one year from the beginning of the pandemic, Fig. 6. Since the appearance of SARS-CoV-2, scientists have tried different ways to develop vaccines and the most significant ones include modified mRNA encoding S-protein (Moderna and BioNTech), the replication-defective viral vector containing the S-protein sequence (Ad5-nCov-CanSino, ChAdOx1 based AZD1222-A-AstraZeneca, GRAd-COV2-Reithera), inactivated pathogenic SARS-CoV-2 (SinoVac, SinoPharm), and recombinant viral subunit proteins (entire S-protein or RBD), Table 1. Among the 10 billion doses delivered, so far the mRNA vaccines from BioNTech and Moderna as well as the viral vector-based vaccine from AstraZeneca and the inactivated vaccines from Sinovac and Sinopharm occupy more than 95% of the market.

**Table 1:** Top 20 COVID-19 vaccines in use.

No.	Name	Produced doses(K)	Percentage (%)	Type	Administration	Common side effects
1	Pfizer BioNTech - Comirnaty	5,341,277	28.55	mRNA, modified	2 doses, 3 weeks apart	Pain at the injection site, Fatigue, Headache, Chills, Myalgia
2	Moderna - Spikevax	3,229,743	17.26	mRNA, modified	2 doses, 1 month apart	Injection site pain/swelling/erythema, Fatigue, Headache, Myalgia, Arthralgia, Nausea/vomiting, Axillary swelling/tenderness, Fever, Immune thrombotic thrombocytopenia
3	AstraZeneca - Vaxzevria	2,142,294	11.45	Viral vector	2 doses, 8–12 weeks apart	Redness at the injection site, Vomiting, Diarrhoea, Fever, Swelling, Low levels of blood platelets
4	SII - Covishield	1,675,068	8.95	Viral vector	2 doses, 8–12 weeks apart	Redness at the injection site, Vomiting, Diarrhoea, Fever, Swelling, Low levels of blood platelets
5	Sinovac - CoronaVac	1,405,002	7.51	Inactivated virus	2 doses, 4 weeks apart	Injection site pain/swelling/erythema, Fatigue, Headache, Myalgia, Arthralgia, Nausea, Diarrhea, Cough, Chill, Pruritus, Loss of appetite, Rhinorrhea, Sore throat, Nasal congestion, Abdominal pain
6	Janssen - Ad26.COV 2-S	1,354,644	7.24	Viral vector	single dose	Injection site pain/swelling/redness, Headache, Fatigue, Myalgia, Nausea, Coughing, Joint pain, Fever, Chills, Immune thrombotic thrombocytopenia
7	Novavax-NUVAXOVID	927,553	4.96	Protein subunit	2 doses, 3 weeks apart	Injection site pain/tenderness, Fatigue, Headache, Myalgia, Joint pain
8	Beijing CNBG - BBIBP-CorV	852,614	4.56	Inactivated virus	2 doses, 3–4 weeks apart	Injection site pain, Fatigue, Lethargy, Tenderness, Fever
9	Bharat - Covaxin	384,400	2.05	Inactivated virus	2 doses, 4 weeks apart	Injection site pain/swelling, Fever, Headaches, Irritability
10	Shifa - COVIran Barakat	350,000	1.87	Inactivated virus	2 doses, 4 weeks apart	N.A.
11	Medicago - VLP	304,000	1.63	Virus-like particles	2 doses, 3 weeks apart	Injection site pain/swelling, Chills, Fatigue, Joint aches, Headache, Mild fever, Muscle aches, Nasal congestionSore throat, Cough, Nausea, Diarrhea
12	Sanofi GSK - Vidprevtyn	302,880	1.62	Protein subunit	2 doses, 3 weeks apart	N.A.
13	Gamaleya - Gam-Covid-Vac	270,116	1.44	Viral vector	2 doses, 3 weeks apart	Injection site pain/swelling/erythema, Fatigue, Headache, Myalgia, Arthralgia, Nausea/vomiting, Axillary swelling/tenderness, Fever,
14	Curevac - CVnCoV	79,780	0.43	mRNA, unmodified	2 doses, 4 weeks apart	Fatigue, Headache, Chills, Muscle pain, Fever
15	CanSino - Convidecia	28,730	0.15	Viral vector	single dose	Headaches, Fatigue, Injection site reactions
16	Finlay - Soberana-02	26,200	0.14	Conjugate vaccine	2 doses, 4 weeks apart	Injection site pain, Inflammation at the injection site, General discomfort
17	Vaxine-SpikoGen	18,000	0.10	Recombinant protein	2 doses, 3 weeks apart	Injection site pain/erythema/swelling/induration, Axillary swelling/tenderness ipsilateral to the side of injection, Fever, Headache, Fatigue, Myalgia, Arthralgia, Nausea, Vomiting, Chills
18	Gamaleya - Sputnik-Light	5,835	0.03	Viral vector	single dose	Mild pain at the injection site, Fever, Headaches, Fatigue, Muscle aches
19	Chumakov - Covi-Vac	3,012	0.02	Inactivated virus	2 doses, 2 weeks apart	N.A.
20	Valneva - VLA2001	2,232	0.01	Inactivated virus	2 doses, 4 weeks apart	Redness at the injection site, Vomiting, Diarrhoea, Fever, Swelling, Less side effects

References cite: [170, 174].

Due to the emergency of the COVID-19 pandemic, scientists did have the minimum sufficient time to evaluate the effectiveness of vaccines developed, and there is large scope for improvement. For example, the interval between doses (21 days for Pfizer; 28 days for Moderna) seems too short, and understanding of the short-lived antibody response (6 months) is still elusive. The cross-reactivity with SARS and MERS might suggest the possibility of a universal pan-coronavirus vaccine. More, as in other vaccinations, there is the possibility that specific commensal microbiota, helminths, nutrients (bile acids, butyrates) or antibiotics, not to mention an immune-suppressive status, might impair the immune response. So far, there are two most popular methods, antibody levels and protection from infection in the real world, to evaluate the effectiveness of a vaccine. Clearly, the antibody is a good predictor and there are epidemiological data supporting a good correlation between the antibody level and disease susceptibility [153], especially considering the complexity of antibody classes and their kinetics. Since the vaccines can only provide a reduction in severity, there is no good model and specifics to quantitatively analyze and accurately determine the protectability.

We have previously hypothesized that “there are many types or subtypes of coronavirus” -or variants. Thus, if vaccines directly targeting SARS-CoV-2 prove to be difficult to develop, the Edward Jenner approach should be considered [154]. It has been noted that a subset of T cells primed against seasonal coronaviruses cross-react with SARS-CoV-2, and this is believed that it may contribute to clinical protection, particularly in early life. The coronaviruses belong to a family of enveloped single-stranded positive-sense RNA viruses. Available information on cellular immunity to other human coronaviruses (HCoVs), especially those causing the common cold, could be valuable for elucidating immunity to SARS-CoV-2. It is estimated that >90% of adults have experienced prior exposure to common cold viruses. Whether the cellular immunity to other coronaviruses such as SARS-CoV-1 last is still a question, though it has been shown that T cell responses can be elicited after 17 years [155]. Sustained T cell responses have been seen in some patients infected with MERS, though remain to be verified with longitudinal studies in more patients. Considering the wide distribution of horseshoe bats in Southeast Asia and the low SARS-CoV-2 infection rate in the area (3.1% in Southeast Asia, 14.9% in the Americas, and 22.5% In Europe according to the data on WHO COVID-19 Dashboard as of February 2022), it is suggestive that some bat coronavirus(es) may provide natural immunity to native residents. As Edward Jenner did with the cowpox virus to protect humans from smallpox, we may try to identify a bat coronavirus to protect humans from SARS-CoV-2.

A successful vaccine relies on various factors such as the identification of the effective epitopes or viral components, the delivery vectors, proper adjuvant, administration routes and the physical and medical conditions of the recipients [156]. Even if we have effective vaccines, the vaccination rate in a given time, social acceptability/resistance, and inadequate social distance may allow the virus to present in a population for sufficiently long to mutate [157]. As it stands at the moment, none of the available vaccine formulations seem to be capable of completely preventing virus infection, at least when it comes to highly infective variants such as the Omicron variant. What needs to be considered under these circumstances with often only partial immune protective conditions is that the virus is subjected to an unfavorable situation that may force the virus to mutate.

## What will follow Omicron

Among all the variants of SARS-CoV-2, Omicron brings the most worries, confusion, and expectations. The fast transmission rate of the Omicron variant has raised serious concerns among epidemiologists, politicians, and disease control experts since it was first reported from South Africa on November 24, 2021 [158]. Many factors contribute to the fast spread. It is possible that the virus begins to spread shortly after the initial infection and long before the appearance of symptoms. Omicron is approximately 10 times more contagious than wild-type SARS-CoV-2 or 2.8 times as infectious as Delta. The newly emerged new Omicron BA.2 is even more contagious [159]. Mutation analysis of Omicron and its BA.2 variants indicates that their spike proteins carry large amounts of mutations far more than the previous VoC, Fig. 1. Of the mutations, D614G is a well-known mutation that confers enhanced infectivity by multiple mechanisms including a bi-modular impact on the stability of spike trimer [160, 161]; the mutations in K417 and E484 of the RBD region were believed to alter spike affinity to ACE2 [162]; the N501Y mutation had been shown to change virus tropism by endowing cross-species transmission to mice [163], therefore creating a potential intermediate host that helps virus spreading [164]. Indeed, latest studies identified Omicron infection in rats and mice [165–167], supporting the zoonotic transmission of the new SARS-CoV-2 variant of concerns. It is expected that the mutations in the Omicron RBD together altered the affinity to ACE2, however, protein structural analysis did not find a higher affinity [168]. The contributing factors to the highly contagious nature remain a mystery. Nevertheless, the high ability to spread and the seemingly less pathogenicity have ignited the hope of herd immunity and the ending of the pandemic. The question is whether Omicron is indeed less pathogenic now but can acquire increased pathogenicity through further mutations. There is no guarantee that the next variant will be milder. The most worrisome is the appearance of Deltacron, which has the backbone of the Delta variant and the spike of Omicron. Information on the transmission speed and pathogenicity of Deltacron is urgently needed.

There is a lot of debate regarding the effectiveness of the existing vaccines against Omicron. Almost all vaccine-induced immunity could be invaded by the Omicron variant. Due to their lower ability to induce antibodies, the inactivated vaccines are believed to be not as effective in providing protection against infection. Scientists are waiting for the recent information on the disease severity of patients infected with Omicron from China mainland, where nearly 90% have received inactivated vaccines, and that from Hong Kong, where the majority of people are immunized with the RNA vaccines. It should be noted that a multinational study showed that recipients immunized first with inactivated vaccine followed by an RNA vaccine showed the highest RBD specific antibody and Omicron specific T cells as compared to two immunizations with a single vaccine type. May inactivated vaccine induces more T helper cells due to being presented by MHC class II, Fig. 6? It has been reported that heterologous immunization with inactivated vaccine followed by an mRNA booster elicits strong humoral and cellular immune responses against the SARS-CoV-2 Omicron variant [169].

Finally, how will the COVID-19 pandemic end? Will Omicron be the last variant? If not, what properties will the next variant have? The biggest question is whether COVID-19 will become endemic. We just hope that, with the immunity built up by vaccination and infection in the population, the endemic is not as deadly. Clearly, we have to learn the new routines of SARS-CoV-2.

## References

[1] Chen J, Lu H, Melino G, Boccia S, Piacentini M, Ricciardi W (2020). COVID-19 infection: the China and Italy perspectives. Cell Death Dis.

[2] Ackermann M, Anders HJ, Bilyy R, Bowlin GL, Daniel C, De Lorenzo R (2021). Patients with COVID-19: in the dark-NETs of neutrophils. Cell Death Differ.

[3] Goubet AG, Dubuisson A, Geraud A, Danlos FX, Terrisse S, Silva CAC (2021). Prolonged SARS-CoV-2 RNA virus shedding and lymphopenia are hallmarks of COVID-19 in cancer patients with poor prognosis. Cell Death Differ.

[4] Matsuyama T, Kubli SP, Yoshinaga SK, Pfeffer K, Mak TW (2020). An aberrant STAT pathway is central to COVID-19. Cell Death Differ.

[5] Matsuyama T, Yoshinaga SK, Shibue K, Mak TW (2021). Comorbidity-associated glutamine deficiency is a predisposition to severe COVID-19. Cell Death Differ.

[6] Verkhratsky A, Li Q, Melino S, Melino G, Shi Y (2020). Can COVID-19 pandemic boost the epidemic of neurodegenerative diseases?. Biol Direct.

[7] Murray CJL, Piot P (2021). The potential future of the COVID-19 pandemic: will SARS-CoV-2 become a recurrent seasonal infection?. JAMA..

[8] Buonvino S, Melino S (2020). New Consensus pattern in Spike CoV-2: potential implications in coagulation process and cell-cell fusion. Cell Death Disco.

[9] Colson P, Fournier PE, Delerce J, Million M, Bedotto M, Houhamdi L, et al. Culture and identification of a "Deltamicron" SARS-CoV-2 in a three cases cluster in southern France. J Med Virol. 2022. Online ahead of print. 10.1002/jmv.27789 PMC9088576 35467028

[10] Forni G, Mantovani A (2021). Covid-19 Commission of Accademia Nazionale dei Lincei R. COVID-19 vaccines: where we stand and challenges ahead. Cell Death Differ.

[11] Lin L, Wang Y, Li Q, Hu M, Shi Y (2022). Novel SARS-CoV-2 therapeutic targets: RNA proofreading complex and virus-induced senescence. Cell Death Differ.

[12] Shi Y, Wang Y, Shao C, Huang J, Gan J, Huang X (2020). COVID-19 infection: the perspectives on immune responses. Cell Death Differ.

[13] Mauriello A, Scimeca M, Amelio I, Massoud R, Novelli A, Di Lorenzo F (2021). Thromboembolism after COVID-19 vaccine in patients with preexisting thrombocytopenia. Cell Death Dis.

[14] Telenti A, Arvin A, Corey L, Corti D, Diamond MS, Garcia-Sastre A (2021). After the pandemic: perspectives on the future trajectory of COVID-19. Nature..

[15] Sacco G, Briere O, Asfar M, Guerin O, Berrut G, Annweiler C (2020). Symptoms of COVID-19 among older adults: a systematic review of biomedical literature. Geriatr. Psychol Neuropsychiatr Vieil.

[16] Dehingia N, Raj A (2021). Sex differences in COVID-19 case fatality: do we know enough? Lancet. Glob Health.

[17] Flaherty GT, Hession P, Liew CH, Lim BCW, Leong TK, Lim V (2020). COVID-19 in adult patients with pre-existing chronic cardiac, respiratory and metabolic disease: a critical literature review with clinical recommendations. Trop Dis Travel Med Vaccines.

[18] Karim SSA, Karim QA (2021). Omicron SARS-CoV-2 variant: a new chapter in the COVID-19 pandemic. Lancet..

[19] Kozlov M (2022). Omicron’s feeble attack on the lungs could make it less dangerous. Nature..

[20] Wolter N, Jassat W, Walaza S, Welch R, Moultrie H, Groome M (2022). Early assessment of the clinical severity of the SARS-CoV-2 omicron variant in South Africa: a data linkage study. Lancet..

[21] Irving AT, Ahn M, Goh G, Anderson DE, Wang LF (2021). Lessons from the host defences of bats, a unique viral reservoir. Nature..

[22] Burki TK (2022). Omicron variant and booster COVID-19 vaccines. Lancet Respir Med.

[23] Junqueira C, Crespo A, Ranjbar S, de Lacerda LB, Lewandrowski M, Ingber J, et al. FcgammaR-mediated SARS-CoV-2 infection of monocytes activates inflammation. Nature. 2022. Online ahead of print. 10.1038/s41586-022-04702-4 PMC10071495 35385861

[24] Moncunill G, Mayor A, Santano R, Jimenez A, Vidal M, Tortajada M (2021). SARS-CoV-2 seroprevalence and antibody kinetics among health care workers in a spanish hospital after 3 months of follow-up. J Infect Dis.

[25] Zipeto D, Palmeira JDF, Arganaraz GA, Arganaraz ER (2020). ACE2/ADAM17/TMPRSS2 interplay may be the main risk factor for COVID-19. Front Immunol.

[26] Callaway E (2022). Scientists deliberately gave people COVID - here’s what they learnt. Nature..

[27] Schulte-Schrepping J, Reusch N, Paclik D, Bassler K, Schlickeiser S, Zhang B (2020). Severe COVID-19 is marked by a dysregulated myeloid cell compartment. Cell..

[28] Zhang Q, Bastard P, Liu Z, Le Pen J, Moncada-Velez M, Chen J, et al. Inborn errors of type I IFN immunity in patients with life-threatening COVID-19. Science. 2020;370:eabd4570. 10.1126/science.abd4570 PMC7857407 32972995

[29] Ellinghaus D, Degenhardt F, Bujanda L, Buti M, Albillos A, Invernizzi P (2020). Genomewide association study of severe Covid-19 with respiratory failure. N Engl J Med.

[30] Delorey TM, Ziegler CGK, Heimberg G, Normand R, Yang Y, Segerstolpe A (2021). COVID-19 tissue atlases reveal SARS-CoV-2 pathology and cellular targets. Nature..

[31] Schultze JL, Aschenbrenner AC (2021). COVID-19 and the human innate immune system. Cell.

[32] Salvi V, Nguyen HO, Sozio F, Schioppa T, Gaudenzi C, Laffranchi M, et al. SARS-CoV-2-associated ssRNAs activate inflammation and immunity via TLR7/8. JCI Insight. 2021;6:e150542. 10.1172/jci.insight.150542 PMC8492321 34375313

[33] van der Made CI, Simons A, Schuurs-Hoeijmakers J, van den Heuvel G, Mantere T, Kersten S (2020). Presence of genetic variants among young men with severe COVID-19. JAMA..

[34] Lu Q, Liu J, Zhao S, Gomez Castro MF, Laurent-Rolle M, Dong J (2021). SARS-CoV-2 exacerbates proinflammatory responses in myeloid cells through C-type lectin receptors and Tweety family member 2. Immunity..

[35] Lempp FA, Soriaga LB, Montiel-Ruiz M, Benigni F, Noack J, Park YJ (2021). Lectins enhance SARS-CoV-2 infection and influence neutralizing antibodies. Nature..

[36] Stravalaci M, Pagani I, Paraboschi EM, Pedotti M, Doni A, Scavello F (2022). Recognition and inhibition of SARS-CoV-2 by humoral innate immunity pattern recognition molecules. Nat Immunol.

[37] Chiodo F, Bruijns S, Rodriguez E, Eveline Li RJ, Molinaro A, Silipo A, et al. Novel ACE2-independent carbohydrate-binding of SARS-CoV-2 spike protein to host lectins and lung microbiota. Preprint at https://www.biorxiv.org/content/10.1101/2020.05.13.092478v1. 2020.

[38] Muus C, Luecken MD, Eraslan G, Sikkema L, Waghray A, Heimberg G (2021). Single-cell meta-analysis of SARS-CoV-2 entry genes across tissues and demographics. Nat Med.

[39] Grant RA, Morales-Nebreda L, Markov NS, Swaminathan S, Querrey M, Guzman ER (2021). Circuits between infected macrophages and T cells in SARS-CoV-2 pneumonia. Nature..

[40] van Beek AA, Van den Bossche J, Mastroberardino PG, de Winther MPJ, Leenen PJM (2019). Metabolic alterations in aging macrophages: ingredients for inflammaging?. Trends Immunol.

[41] Locati M, Curtale G, Mantovani A (2020). Diversity, mechanisms, and significance of macrophage plasticity. Annu Rev Pathol.

[42] Xue J, Schmidt SV, Sander J, Draffehn A, Krebs W, Quester I (2014). Transcriptome-based network analysis reveals a spectrum model of human macrophage activation. Immunity..

[43] Mantovani A, Netea MG (2020). Trained innate immunity, epigenetics, and Covid-19. N Engl J Med.

[44] Habibzadeh F, Sajadi MM, Chumakov K, Yadollahie M, Kottilil S, Simi A (2021). COVID-19 infection among women in iran exposed vs unexposed to children who received attenuated poliovirus used in oral polio vaccine. JAMA Netw Open.

[45] Wendisch D, Dietrich O, Mari T, von Stillfried S, Ibarra IL, Mittermaier M (2021). SARS-CoV-2 infection triggers profibrotic macrophage responses and lung fibrosis. Cell..

[46] Kramer B, Knoll R, Bonaguro L, ToVinh M, Raabe J, Astaburuaga-Garcia R (2021). Early IFN-alpha signatures and persistent dysfunction are distinguishing features of NK cells in severe COVID-19. Immunity..

[47] Witkowski M, Tizian C, Ferreira-Gomes M, Niemeyer D, Jones TC, Heinrich F (2021). Untimely TGFbeta responses in COVID-19 limit antiviral functions of NK cells. Nature..

[48] Bernardes JP, Mishra N, Tran F, Bahmer T, Best L, Blase JI (2020). Longitudinal multi-omics analyses identify responses of megakaryocytes, erythroid cells, and plasmablasts as hallmarks of severe COVID-19. Immunity..

[49] Georg P, Astaburuaga-Garcia R, Bonaguro L, Brumhard S, Michalick L, Lippert LJ (2022). Complement activation induces excessive T cell cytotoxicity in severe COVID-19. Cell..

[50] Bottazzi B, Doni A, Garlanda C, Mantovani A (2010). An integrated view of humoral innate immunity: pentraxins as a paradigm. Annu Rev Immunol.

[51] Garlanda C, Bottazzi B, Magrini E, Inforzato A, Mantovani A (2018). PTX3, a humoral pattern recognition molecule, in innate immunity, tissue repair, and cancer. Physiol Rev.

[52] Brunetta E, Folci M, Bottazzi B, De Santis M, Gritti G, Protti A (2021). Macrophage expression and prognostic significance of the long pentraxin PTX3 in COVID-19. Nat Immunol.

[53] Gritti G, Raimondi F, Bottazzi B, Ripamonti D, Riva I, Landi F (2021). Siltuximab downregulates interleukin-8 and pentraxin 3 to improve ventilatory status and survival in severe COVID-19. Leukemia..

[54] Gutmann C, Takov K, Burnap SA, Singh B, Ali H, Theofilatos K (2021). SARS-CoV-2 RNAemia and proteomic trajectories inform prognostication in COVID-19 patients admitted to intensive care. Nat Commun.

[55] Hansen FC, Nadeem A, Browning KL, Campana M, Schmidtchen A, van der Plas MJA. Differential internalization of thrombin-derived host defense peptides into monocytes and macrophages. J Innate Immun. 2021:1–15. 10.1159/000520831 PMC9485985 34937021

[56] Schirinzi A, Pesce F, Laterza R, D’Alise MG, Lovero R, Fontana A (2021). Pentraxin 3: potential prognostic role in SARS-CoV-2 patients admitted to the emergency department. J Infect.

[57] Phetsouphanh C, Darley DR, Wilson DB, Howe A, Munier CML, Patel SK (2022). Immunological dysfunction persists for 8 months following initial mild-to-moderate SARS-CoV-2 infection. Nat Immunol.

[58] Risitano AM, Mastellos DC, Huber-Lang M, Yancopoulou D, Garlanda C, Ciceri F (2020). Complement as a target in COVID-19?. Nat Rev Immunol.

[59] Mastellos DC, Pires da Silva BGP, Fonseca BAL, Fonseca NP, Auxiliadora-Martins M, Mastaglio S (2020). Complement C3 vs C5 inhibition in severe COVID-19: early clinical findings reveal differential biological efficacy. Clin Immunol.

[60] Mastaglio S, Ruggeri A, Risitano AM, Angelillo P, Yancopoulou D, Mastellos DC (2020). The first case of COVID-19 treated with the complement C3 inhibitor AMY-101. Clin Immunol.

[61] Rambaldi A, Gritti G, Mico MC, Frigeni M, Borleri G, Salvi A (2020). Endothelial injury and thrombotic microangiopathy in COVID-19: treatment with the lectin-pathway inhibitor narsoplimab. Immunobiology..

[62] Bumiller-Bini V, de Freitas Oliveira-Tore C, Carvalho TM, Kretzschmar GC, Goncalves LB, Alencar NM (2021). MASPs at the crossroad between the complement and the coagulation cascades - the case for COVID-19. Genet Mol Biol..

[63] Flude BM, Nannetti G, Mitchell P, Compton N, Richards C, Heurich M, et al. Targeting the complement serine protease MASP-2 as a therapeutic strategy for coronavirus infections. Viruses. 2021;13:312. 10.3390/v13020312 PMC7923061 33671334

[64] Junqueira C, Crespo A, Ranjbar S, Lewandrowski M, Ingber J, de Lacerda LB, et al. SARS-CoV-2 infects blood monocytes to activate NLRP3 and AIM2 inflammasomes, pyroptosis and cytokine release. Res Sq. 2021.

[65] Sefik E, Qu R, Junqueira C, Kaffe E, Mirza H, Zhao J, et al. Inflammasome activation in infected macrophages drives COVID-19 pathology. Nature. 2022. Online ahead of print. 10.1038/s41586-022-04802-1 PMC9288243 35483404

[66] Lucas C, Wong P, Klein J, Castro TBR, Silva J, Sundaram M (2020). Longitudinal analyses reveal immunological misfiring in severe COVID-19. Nature..

[67] Zheng M, Karki R, Williams EP, Yang D, Fitzpatrick E, Vogel P (2021). TLR2 senses the SARS-CoV-2 envelope protein to produce inflammatory cytokines. Nat Immunol.

[68] Rodrigues TS, de Sá KSG, Ishimoto AY, Becerra A, Oliveira S, Almeida L, et al. Inflammasomes are activated in response to SARS-CoV-2 infection and are associated with COVID-19 severity in patients. J Exp Med. 2021;218:e20201707. 10.1084/jem.20201707 PMC7684031 33231615

[69] Agrati C, Sacchi A, Bordoni V, Cimini E, Notari S, Grassi G (2020). Expansion of myeloid-derived suppressor cells in patients with severe coronavirus disease (COVID-19). Cell Death Differ.

[70] Knopf J, Leppkes M, Schett G, Herrmann M, Muñoz LE (2019). Aggregated NETs sequester and detoxify extracellular histones. Front Immunol.

[71] Schauer C, Janko C, Munoz LE, Zhao Y, Kienhöfer D, Frey B (2014). Aggregated neutrophil extracellular traps limit inflammation by degrading cytokines and chemokines. Nat Med.

[72] Leppkes M, Knopf J, Naschberger E, Lindemann A, Singh J, Herrmann I (2020). Vascular occlusion by neutrophil extracellular traps in COVID-19. EBioMedicine..

[73] Yaykasli KO, Schauer C, Muñoz LE, Mahajan A, Knopf J, Schett G, et al. Neutrophil extracellular trap-driven occlusive diseases. Cells. 2021;10:2201. 10.3390/cells10092208 PMC8466545 34571857

[74] Zuo Y, Yalavarthi S, Shi H, Gockman K, Zuo M, Madison JA, et al. Neutrophil extracellular traps in COVID-19. JCI insight. 2020;5:e138999. 10.1172/jci.insight.138999 PMC7308057 32329756

[75] Arcanjo A, Logullo J, Menezes CCB, de Souza Carvalho Giangiarulo TC, Dos Reis MC, de Castro GMM (2020). The emerging role of neutrophil extracellular traps in severe acute respiratory syndrome coronavirus 2 (COVID-19). Sci Rep.

[76] Middleton EA, He XY, Denorme F, Campbell RA, Ng D, Salvatore SP (2020). Neutrophil extracellular traps contribute to immunothrombosis in COVID-19 acute respiratory distress syndrome. Blood..

[77] Veras FP, Pontelli MC, Silva CM, Toller-Kawahisa JE, de Lima M, Nascimento DC, et al. SARS-CoV-2-triggered neutrophil extracellular traps mediate COVID-19 pathology. J Exp Med. 2020;217:e20201129. 10.1084/jem.20201129 PMC7488868 32926098

[78] Petito E, Falcinelli E, Paliani U, Cesari E, Vaudo G, Sebastiano M (2021). Association of neutrophil activation, more than platelet activation, with thrombotic complications in coronavirus disease 2019. J Infect Dis.

[79] Skendros P, Mitsios A, Chrysanthopoulou A, Mastellos DC, Metallidis S, Rafailidis P (2020). Complement and tissue factor-enriched neutrophil extracellular traps are key drivers in COVID-19 immunothrombosis. J Clin Invest.

[80] Bilyy R, Bila G, Vishchur O, Vovk V, Herrmann M. Neutrophils as main players of immune response towards nondegradable nanoparticles. Nanomaterials. 2020;10:1273. 10.3390/nano10071273 PMC7408411 32610567

[81] Liu J, Li J, Arnold K, Pawlinski R, Key NS (2020). Using heparin molecules to manage COVID-2019. Res Pract thrombosis Haemost.

[82] Weber AG, Chau AS, Egeblad M, Barnes BJ, Janowitz T (2020). Nebulized in-line endotracheal dornase alfa and albuterol administered to mechanically ventilated COVID-19 patients: a case series. Mol Med.

[83] Desilles JP, Gregoire C, Le Cossec C, Lambert J, Mophawe O, Losser MR (2020). Efficacy and safety of aerosolized intra-tracheal dornase alfa administration in patients with SARS-CoV-2-induced acute respiratory distress syndrome (ARDS): a structured summary of a study protocol for a randomised controlled trial. Trials..

[84] Adrover JM, Carrau L, Daßler-Plenker J, Bram Y, Chandar V, Houghton S, et al. Disulfiram inhibits neutrophil extracellular trap formation and protects rodents from acute lung injury and SARS-CoV-2 infection. JCI insight. 2022;7:e157342. 10.1172/jci.insight.157342 PMC8983145 35133984

[85] Elliott W, Jr., Guda MR, Asuthkar S, Teluguakula N, Prasad DVR, Tsung AJ, et al. PAD inhibitors as a potential treatment for SARS-CoV-2 immunothrombosis. Biomedicines. 2021;9:1867. 10.3390/biomedicines9121867 PMC8698348 34944683

[86] MacMicking JD (2012). Interferon-inducible effector mechanisms in cell-autonomous immunity. Nat Rev Immunol.

[87] Tan X, Sun L, Chen J, Chen ZJ (2018). Detection of microbial infections through innate immune sensing of nucleic acids. Annu Rev Microbiol.

[88] Xue W, Ding C, Qian K, Liao Y (2021). The interplay between coronavirus and type I IFN response. Front Microbiol.

[89] Lei X, Dong X, Ma R, Wang W, Xiao X, Tian Z (2020). Activation and evasion of type I interferon responses by SARS-CoV-2. Nat Commun.

[90] Lee JS, Shin EC (2020). The type I interferon response in COVID-19: implications for treatment. Nat Rev Immunol.

[91] Humphries F, Shmuel-Galia L, Jiang Z, Wilson R, Landis P, Ng SL, et al. A diamidobenzimidazole STING agonist protects against SARS-CoV-2 infection. Sci Immunol. 2021;6:eabi9002. 10.1126/sciimmunol.abi9002 PMC8158975 34010139

[92] Li M, Ferretti M, Ying B, Descamps H, Lee E, Dittmar M, et al. Pharmacological activation of STING blocks SARS-CoV-2 infection. Sci Immunol. 2021;6. 10.1126/sciimmunol.abi9007 PMC10021026 34010142

[93] Bernard NJ (2021). A STING in the tail for SARS-CoV-2. Nat Immunol.

[94] Zhao Y, Sui L, Wu P, Wang W, Wang Z, Yu Y (2021). A dual-role of SARS-CoV-2 nucleocapsid protein in regulating innate immune response. Signal Transduct Target Ther.

[95] Ren H, Ma C, Peng H, Zhang B, Zhou L, Su Y (2021). Micronucleus production, activation of DNA damage response and cGAS-STING signaling in syncytia induced by SARS-CoV-2 infection. Biol Direct.

[96] Zhou Z, Zhang X, Lei X, Xiao X, Jiao T, Ma R (2021). Sensing of cytoplasmic chromatin by cGAS activates innate immune response in SARS-CoV-2 infection. Signal Transduct Target Ther.

[97] Rui Y, Su J, Shen S, Hu Y, Huang D, Zheng W (2021). Unique and complementary suppression of cGAS-STING and RNA sensing- triggered innate immune responses by SARS-CoV-2 proteins. Signal Transduct Target Ther.

[98] Denning DW, Kilcoyne A, Ucer C (2020). Non-infectious status indicated by detectable IgG antibody to SARS-CoV-2. Br Dent J.

[99] Long QX, Liu BZ, Deng HJ, Wu GC, Deng K, Chen YK (2020). Antibody responses to SARS-CoV-2 in patients with COVID-19. Nat Med.

[100] Nakano Y, Kurano M, Morita Y, Shimura T, Yokoyama R, Qian C (2021). Time course of the sensitivity and specificity of anti-SARS-CoV-2 IgM and IgG antibodies for symptomatic COVID-19 in Japan. Sci Rep.

[101] Moura AD, da Costa HHM, Correa VA, de SLAK, Lindoso JAL, De Gaspari E (2021). Assessment of avidity related to IgG subclasses in SARS-CoV-2 Brazilian infected patients. Sci Rep.

[102] Sterlin D, Mathian A, Miyara M, Mohr A, Anna F, Claer L, et al. IgA dominates the early neutralizing antibody response to SARS-CoV-2. Sci Transl Med. 2021;13:eabd2223. 10.1126/scitranslmed.abd2223 PMC7857408 33288662

[103] Zheng HY, Zhang M, Yang CX, Zhang N, Wang XC, Yang XP (2020). Elevated exhaustion levels and reduced functional diversity of T cells in peripheral blood may predict severe progression in COVID-19 patients. Cell Mol Immunol.

[104] Rydyznski Moderbacher C, Ramirez SI, Dan JM, Grifoni A, Hastie KM, Weiskopf D (2020). Antigen-specific adaptive immunity to SARS-CoV-2 in acute COVID-19 and associations with age and disease severity. Cell..

[105] Remy KE, Mazer M, Striker DA, Ellebedy AH, Walton AH, Unsinger J, et al. Severe immunosuppression and not a cytokine storm characterizes COVID-19 infections. JCI Insight. 2020;5:e140329. 10.1172/jci.insight.140329 PMC7526441 32687484

[106] Nielsen SCA, Yang F, Jackson KJL, Hoh RA, Roltgen K, Jean GH (2020). Human B cell clonal expansion and convergent antibody responses to SARS-CoV-2. Cell Host Microbe.

[107] Lucas C, Klein J, Sundaram ME, Liu F, Wong P, Silva J (2021). Delayed production of neutralizing antibodies correlates with fatal COVID-19. Nat Med.

[108] Li K, Huang B, Wu M, Zhong A, Li L, Cai Y (2020). Dynamic changes in anti-SARS-CoV-2 antibodies during SARS-CoV-2 infection and recovery from COVID-19. Nat Commun.

[109] Kaneko N, Kuo HH, Boucau J, Farmer JR, Allard-Chamard H, Mahajan VS (2020). Loss of Bcl-6-expressing T follicular helper cells and germinal centers in COVID-19. Cell.

[110] Van Cleemput J, van Snippenberg W, Lambrechts L, Dendooven A, D’Onofrio V, Couck L (2021). Organ-specific genome diversity of replication-competent SARS-CoV-2. Nat Commun.

[111] Baize S, Leroy EM, Georges-Courbot MC, Capron M, Lansoud-Soukate J, Debre P (1999). Defective humoral responses and extensive intravascular apoptosis are associated with fatal outcome in Ebola virus-infected patients. Nat Med.

[112] Estaquier J, Idziorek T, de Bels F, Barre-Sinoussi F, Hurtrel B, Aubertin AM (1994). Programmed cell death and AIDS: significance of T-cell apoptosis in pathogenic and nonpathogenic primate lentiviral infections. Proc Natl Acad Sci USA.

[113] Monceaux V, Estaquier J, Février M, Cumont MC, Rivière Y, Aubertin AM (2003). Extensive apoptosis in lymphoid organs during primary SIV infection predicts rapid progression towards AIDS. Aids..

[114] André S, Picard M, Cezar R, Roux-Dalvai F, Alleaume-Butaux A, Soundaramourty C, et al. T cell apoptosis characterizes severe Covid-19 disease. Cell Death Differ. 2022:1–14. Online ahead of print. 10.1038/s41418-022-00936-x PMC8782710 35066575

[115] Butler D, Mozsary C, Meydan C, Foox J, Rosiene J, Shaiber A (2021). Shotgun transcriptome, spatial omics, and isothermal profiling of SARS-CoV-2 infection reveals unique host responses, viral diversification, and drug interactions. Nat Commun.

[116] Yin W, Napoleon MA, Suder EL, Berrigan J, Zhao Q (2021). CD209L/L-SIGN and CD209/DC-SIGN Act as Receptors for SARS-CoV-2. ACS Cent Sci..

[117] Del Valle DM, Kim-Schulze S, Huang HH, Beckmann ND, Nirenberg S, Wang B (2020). An inflammatory cytokine signature predicts COVID-19 severity and survival. Nat Med..

[118] Wiersinga WJ, Rhodes A, Cheng AC, Peacock SJ, Prescott HC (2020). Pathophysiology, transmission, diagnosis, and treatment of coronavirus disease 2019 (COVID-19): a review. JAMA J Am Med Assoc.

[119] Zhou L, Niu Z, Jiang X, Zhang Z, Zheng Y, Wang Z (2020). SARS-CoV-2 targets by the pscRNA profiling of ACE2, TMPRSS2 and furin proteases. iScience.

[120] Bellesi S, Metafuni E, Hohaus S, Maiolo E, Marchionni F, D’Innocenzo S (2020). Increased CD95 (Fas) and PD-1 expression in peripheral blood T lymphocytes in COVID-19 patients. Br J Haematol.

[121] Karki R, Sharma BR, Tuladhar S, Williams EP, Zalduondo L, Samir P (2021). Synergism of TNF-α and IFN-γ triggers inflammatory cell death, tissue damage, and mortality in SARS-CoV-2 infection and cytokine shock syndromes. Cell..

[122] Zhang Z, Zheng Y, Niu Z, Zhang B, Wang C, Yao X (2021). SARS-CoV-2 spike protein dictates syncytium-mediated lymphocyte elimination. Cell Death Differ.

[123] Huang H, He M, Zhang Y, Zhang B, Niu Z, Zheng Y (2020). Identification and validation of heterotypic cell-in-cell structure as an adverse prognostic predictor for young patients of resectable pancreatic ductal adenocarcinoma. Signal Transduct Target Ther.

[124] Su Y, Huang H, Luo T, Zheng Y, Fan J, Ren H (2022). Cell-in-cell structure mediates in-cell killing suppressed by CD44. Cell Disco.

[125] Liang J, Niu Z, Zhang B, Yu X, Zheng Y, Wang C (2021). p53-dependent elimination of aneuploid mitotic offspring by entosis. Cell Death Differ.

[126] Sun Q, Luo T, Ren Y, Florey O, Shirasawa S, Sasazuki T (2014). Competition between human cells by entosis. Cell Res.

[127] Sun Q, Chen W (2022). Cell-in-cell: an emerging player in COVID-19 and immune disorders. Natl Sci Open..

[128] Braga L, Ali H, Secco I, Chiavacci E, Neves G, Goldhill D (2021). Drugs that inhibit TMEM16 proteins block SARS-CoV-2 spike-induced syncytia. Nature..

[129] Zheng Y, Zhou LL, Su Y, Sun Q (2021). Cell fusion in the pathogenesis of COVID-19. Mil Med Res.

[130] Lin L, Li Q, Wang Y, Shi Y (2021). Syncytia formation during SARS-CoV-2 lung infection: a disastrous unity to eliminate lymphocytes. Cell Death Differ..

[131] Ma H, Zhu Z, Lin H, Wang S, Zhang P, Li Y (2021). Pyroptosis of syncytia formed by fusion of SARS-CoV-2 spike and ACE2-expressing cells. Cell Disco.

[132] Meng B, Ferreira IATM, Abdullahi A, Saito A, Kimura I, Yamasoba D, et al. SARS-CoV-2 Omicron spike mediated immune escape, infectivity and cell-cell fusion. Preprint at https://www.biorxiv.org/content/10.1101/2021.12.17.473248v2. 2021.

[133] Willett BJ, Grove J, MacLean OA, Wilkie C, Logan N, Lorenzo GD, et al. The hyper-transmissible SARS-CoV-2 Omicron variant exhibits significant antigenic change, vaccine escape and a switch in cell entry mechanism. Preprint at https://www.medrxiv.org/content/10.1101/2022.01.03.21268111v1. 2022.

[134] Peacock TP, Brown JC, Zhou J, Thakur N, Newman J, Kugathasan R, et al. The SARS-CoV-2 variant, Omicron, shows rapid replication in human primary nasal epithelial cultures and efficiently uses the endosomal route of entry. Preprint at https://www.biorxiv.org/content/10.1101/2021.12.31.474653v1. 2021.

[135] Hamilton SE, Wolkers MC, Schoenberger SP, Jameson SC (2006). The generation of protective memory-like CD8+ T cells during homeostatic proliferation requires CD4+ T cells. Nat Immunol.

[136] Janssen EM, Droin NM, Lemmens EE, Pinkoski MJ, Bensinger SJ, Ehst BD (2005). CD4+ T-cell help controls CD8+ T-cell memory via TRAIL-mediated activation-induced cell death. Nature..

[137] Mazzoni A, Salvati L, Maggi L, Capone M, Vanni A, Spinicci M (2020). Impaired immune cell cytotoxicity in severe COVID-19 is IL-6 dependent. J Clin Invest.

[138] Laforge M, Silvestre R, Rodrigues V, Garibal J, Campillo-Gimenez L, Mouhamad S (2018). The anti-caspase inhibitor Q-VD-OPH prevents AIDS disease progression in SIV-infected rhesus macaques. J Clin Invest.

[139] Sacco K, Castagnoli R, Vakkilainen S, Liu C, Delmonte OM, Oguz C, et al. Immunopathological signatures in multisystem inflammatory syndrome in children and pediatric COVID-19. Nat. Med. 2022. Online ahead of print. 10.1038/s41591-022-01724-3 PMC9119950 35177862

[140] Shi Y, Devadas S, Greeneltch KM, Yin D, Allan Mufson R, Zhou JN (2003). Stressed to death: implication of lymphocyte apoptosis for psychoneuroimmunology. Brain Behav Immun.

[141] Papanicolaou DA, Wilder RL, Manolagas SC, Chrousos GP (1998). The pathophysiologic roles of interleukin-6 in human disease. Ann Intern Med.

[142] Hong M, Zheng J, Ding ZY, Chen JH, Yu L, Niu Y (2013). Imbalance between Th17 and Treg cells may play an important role in the development of chronic unpredictable mild stress-induced depression in mice. Neuroimmunomodulation..

[143] Westfall S, Caracci F, Estill M, Frolinger T, Shen L, Pasinetti GM (2021). Chronic stress-induced depression and anxiety priming modulated by gut-brain-axis immunity. Front Immunol.

[144] Yin D, Tuthill D, Mufson RA, Shi Y (2000). Chronic restraint stress promotes lymphocyte apoptosis by modulating CD95 expression. J Exp Med.

[145] Rajewsky K (2019). The advent and rise of monoclonal antibodies. Nature..

[146] American Academy of Pediatrics Committee on Infectious D, American Academy of Pediatrics Bronchiolitis Guidelines C. (2014). Updated guidance for palivizumab prophylaxis among infants and young children at increased risk of hospitalization for respiratory syncytial virus infection. Pediatrics..

[147] Traggiai E, Becker S, Subbarao K, Kolesnikova L, Uematsu Y, Gismondo MR (2004). An efficient method to make human monoclonal antibodies from memory B cells: potent neutralization of SARS coronavirus. Nat Med.

[148] Sok D, Burton DR (2018). Recent progress in broadly neutralizing antibodies to HIV. Nat Immunol.

[149] Liu L, Wang P, Nair MS, Yu J, Rapp M, Wang Q (2020). Potent neutralizing antibodies against multiple epitopes on SARS-CoV-2 spike. Nature..

[150] Hwang YC, Lu RM, Su SC, Chiang PY, Ko SH, Ke FY (2022). Monoclonal antibodies for COVID-19 therapy and SARS-CoV-2 detection. J Biomed Sci.

[151] Iketani S, Liu L, Guo Y, Liu L, Chan JF, Huang Y (2022). Antibody evasion properties of SARS-CoV-2 Omicron sublineages. Nature..

[152] Cao X (2008). Immunology in China: the past, present and future. Nat Immunol.

[153] Zhu F, Althaus T, Tan CW, Costantini A, Chia WN, Van Vinh Chau N (2022). WHO international standard for SARS-CoV-2 antibodies to determine markers of protection. Lancet Microbe.

[154] Riedel S (2005). Edward Jenner and the history of smallpox and vaccination. Proc Bayl Univ Med Cent.

[155] Le Bert N, Tan AT, Kunasegaran K, Tham CYL, Hafezi M, Chia A (2020). SARS-CoV-2-specific T cell immunity in cases of COVID-19 and SARS, and uninfected controls. Nature.

[156] Tomalka JA, Suthar MS, Deeks SG, Sekaly RP (2022). Fighting the SARS-CoV-2 pandemic requires a global approach to understanding the heterogeneity of vaccine responses. Nat Immunol.

[157] Lobinska G, Pauzner A, Traulsen A, Pilpel Y, Nowak MA (2022). Evolution of resistance to COVID-19 vaccination with dynamic social distancing. Nat Hum Behav.

[158] Kupferschmidt K (2021). Where did ‘weird’ Omicron come from?. Science.

[159] Kupferschmidt K (2021). Where did 'weird' Omicron come from?. Science..

[160] Wang C, Zheng Y, Niu Z, Jiang X, Sun Q (2021). The virological impacts of SARS-CoV-2 D614G mutation. J Mol Cell Biol.

[161] Jiang X, Zhang Z, Wang C, Ren H, Gao L, Peng H (2020). Bimodular effects of D614G mutation on the spike glycoprotein of SARS-CoV-2 enhance protein processing, membrane fusion, and viral infectivity. Signal Transduct Target Ther.

[162] Li Q, Nie J, Wu J, Zhang L, Ding R, Wang H (2021). SARS-CoV-2 501Y.V2 variants lack higher infectivity but do have immune escape. Cell..

[163] Niu Z, Zhang Z, Gao X, Du P, Lu J, Yan B (2021). N501Y mutation imparts cross-species transmission of SARS-CoV-2 to mice by enhancing receptor binding. Signal Transduct Target Ther.

[164] Huang H, Zhu Y, Niu Z, Zhou L, Sun Q (2021). SARS-CoV-2 N501Y variants of concern and their potential transmission by mouse. Cell Death Differ.

[165] Kok KH, Wong SC, Chan WM, Wen L, Chu AW, Ip JD (2022). Co-circulation of two SARS-CoV-2 variant strains within imported pet hamsters in Hong Kong. Emerg Microbes Infect.

[166] Zhang YN, Zhang ZR, Zhang HQ, Li N, Zhang QY, Li XD (2022). Different pathogenesis of SARS-CoV-2 Omicron variant in wild-type laboratory mice and hamsters. Signal Transduct Target Ther.

[167] Shuai H, Chan JF, Yuen TT, Yoon C, Hu JC, Wen L (2021). Emerging SARS-CoV-2 variants expand species tropism to murines. EBioMedicine..

[168] Han P, Li L, Liu S, Wang Q, Zhang D, Xu Z (2022). Receptor binding and complex structures of human ACE2 to spike RBD from omicron and delta SARS-CoV-2. Cell..

[169] Zuo F, Abolhassani H, Du L, Piralla A, Bertoglio F, de Campos-Mata L (2022). Heterologous immunization with inactivated vaccine followed by mRNA-booster elicits strong immunity against SARS-CoV-2 Omicron variant. Nature Communications..

[170] Khare S, Gurry C, Freitas L, Schultz MB, Bach G, Diallo A (2021). GISAID’s role in pandemic response. China CDC Wkly.

[171] Jette CA, Cohen AA, Gnanapragasam PNP, Muecksch F, Lee YE, Huey-Tubman KE (2021). Broad cross-reactivity across sarbecoviruses exhibited by a subset of COVID-19 donor-derived neutralizing antibodies. Cell Rep.

[172] Muecksch F, Weisblum Y, Barnes CO, Schmidt F, Schaefer-Babajew D, Wang Z (2021). Affinity maturation of SARS-CoV-2 neutralizing antibodies confers potency, breadth, and resilience to viral escape mutations. Immunity..

[173] Rino R, Emanuele A, Ida P, Silvia M, Lorena D, Giulio P et al. Anatomy of Omicron neutralizing antibodies in COVID-19 mRNA vaccinees. PREPRINT (Version 1) available at Research Square. 2022. 10.21203/rs.3.rs-1330153/v1.

[174] Latif AA, Mullen JL, Alkuzweny M, Tsueng G, Cano M, Haag E, the Center for Viral Systems Biology, et al. Lineage comparison. outbreak.info. https://outbreak.info/compare-lineages. Accessed 13 April 2022.

